# ASD-Associated *De Novo* Mutations in Five Actin Regulators Show Both Shared and Distinct Defects in Dendritic Spines and Inhibitory Synapses in Cultured Hippocampal Neurons

**DOI:** 10.3389/fncel.2018.00217

**Published:** 2018-08-03

**Authors:** Iryna Hlushchenko, Pushpa Khanal, Amr Abouelezz, Ville O. Paavilainen, Pirta Hotulainen

**Affiliations:** ^1^Minerva Foundation Institute for Medical Research, Helsinki, Finland; ^2^HiLIFE, University of Helsinki, Helsinki, Finland; ^3^Neuroscience Center, University of Helsinki, Helsinki, Finland; ^4^Institute of Biotechnology, University of Helsinki, Helsinki, Finland

**Keywords:** autism spectrum disorder, actin cytoskeleton, dendritic spines, inhibitory synapses, *de novo* point mutations

## Abstract

Many actin cytoskeleton-regulating proteins control dendritic spine morphology and density, which are cellular features often altered in autism spectrum disorder (ASD). Recent studies using animal models show that autism-related behavior can be rescued by either manipulating actin regulators or by reversing dendritic spine density or morphology. Based on these studies, the actin cytoskeleton is a potential target pathway for developing new ASD treatments. Thus, it is important to understand how different ASD-associated actin regulators contribute to the regulation of dendritic spines and how ASD-associated mutations modulate this regulation. For this study, we selected five genes encoding different actin-regulating proteins and induced ASD-associated *de novo* missense mutations in these proteins. We assessed the functionality of the wild-type and mutated proteins by analyzing their subcellular localization, and by analyzing the dendritic spine phenotypes induced by the expression of these proteins. As the imbalance between excitation and inhibition has been suggested to have a central role in ASD, we additionally evaluated the density, size and subcellular localization of inhibitory synapses. Common for all the proteins studied was the enrichment in dendritic spines. ASD-associated mutations induced changes in the localization of α-actinin-4, which localized less to dendritic spines, and for SWAP-70 and SrGAP3, which localized more to dendritic spines. Among the wild-type proteins studied, only α-actinin-4 expression caused a significant change in dendritic spine morphology by increasing the mushroom spine density and decreasing thin spine density. We hypothesized that mutations associated with ASD shift dendritic spine morphology from mushroom to thin spines. An M554V mutation in α-actinin-4 (*ACTN4*) resulted in the expected shift in dendritic spine morphology by increasing the density of thin spines. In addition, we observed a trend toward higher thin spine density with mutations in myosin IXb and SWAP-70. Myosin IIb and myosin IXb expression increased the proportion of inhibitory synapses in spines. The expression of mutated myosin IIb (Y265C), SrGAP3 (E469K), and SWAP-70 (L544F) induced variable changes in inhibitory synapses.

## Introduction

Autism spectrum disorder (ASD) comprises a range of neurological conditions characterized by social deficits, repetitive behaviors, and accompanying comorbidities, including intellectual disability, epilepsy, hyperactivity, and anxiety. ASD has a strong genetic component and almost 1000 genes are currently associated with ASD (SFARI Gene: https://gene.sfari.org/database/human-gene/). Many ASD-associated mutations are rare protein-disrupting *de novo* mutations that arose in the germline. Mutations can be copy-number variants (CNVs) or single-base-pair mutations. Numerous ASD susceptibility genes are involved in regulating the postsynaptic site of glutamatergic synapses (Peça and Feng, 2012; Bourgeron, 2015), the development and maturation of synaptic contacts (Gilman et al., 2011), or synaptic transmission (Li et al., 2014). Most excitatory glutamatergic synapses are located on small dendritic protrusions known as dendritic spines. The formation, maturation, and elimination of dendritic spines lie at the core of synaptic transmission and memory formation (Yang et al., 2009; Roberts et al., 2010). Studies of postmortem human ASD brains revealed an increased spine density, which is—at least in some cases—the result of defective dendritic spine pruning (Tang et al., 2014).

Numerous studies have demonstrated a pivotal role for the actin cytoskeleton in the formation and elimination, motility and stability, and size and shape of dendritic spines (Hotulainen and Hoogenraad, 2010). Actin filaments are polar structures with one end growing more rapidly (the plus or “barbed” end) than the other (the minus or “pointed” end). Constant removal of the actin subunits from the pointed ends and addition at the barbed ends is called actin treadmilling. Synaptic stimulation rapidly changes the actin treadmilling rate (Star et al., 2002; Okamoto et al., 2004; Hlushchenko et al., 2016). The actin treadmilling rate, as well as the three-dimensional organization of actin filaments, are regulated by actin-binding proteins (Hotulainen and Hoogenraad, 2010). Many actin regulators are associated with ASD and these proteins are often involved in the regulation of the structure and function of excitatory synapses (Joensuu et al., 2017). However, our knowledge of whether ASD-associated mutations in actin regulators affect their functions in dendritic spines or synapses is limited.

Recent studies using different animal models have shown that autistic symptoms can be rescued by either manipulating actin regulators or by rescuing dendritic spine density or morphology (Dolan et al., 2013; Duffney et al., 2015). Although it is not yet clear how aberrant dendritic spines and behavioral consequences are connected, these results suggest that actin regulators controlling dendritic spines may play direct causal roles in ASD-related behavior. The social deficits and NMDA receptor hypofunction displayed by *Shank3*-deficient mice were rescued by inhibiting the activity of the actin filament-depolymerizing protein, cofilin, or by activating Rac1, the actin cytoskeleton master-regulator (Duffney et al., 2015). Remarkably, Dolan and colleagues showed that a single administration of p21-activated kinase inhibitor, small molecule FRAX486, was sufficient to recover the phenotype in adult *Fmr1*-knockout mice, demonstrating that a post-diagnostic therapy could be possible for Fragile-X adults (Dolan et al., 2013). p21-activated kinase is a Rac1 effector, which regulates spines through modulation of actin cytoskeleton dynamics. Based on these results, the actin cytoskeleton has recently emerged as a potential new target for new ASD treatments. Thus, it is important to understand how different ASD-associated actin regulators contribute to the regulation of dendritic spines and how ASD-associated mutations modulate this regulation.

When we started this study, the SFARI Gene Database was not yet available. Therefore, to find ASD-associated missense mutations in actin regulators, we relied on the gene and mutation lists published by (Fromer et al., 2014) , which summarized *de novo* mutations in genes associated with different neuropsychiatric diseases. From this list, we selected ASD-associated genes encoding the known actin-regulating proteins: *SRGAP3, TRIO, MYO9B, MYO7B, MYH10, MYO15A, ACTN4, SWAP70, NEB*, and *TTN*. Nebulin (*NEB*) and titin (*TTN*) are giant proteins (nebulin 600–900 kDa, titin up to 4.2 MDa), mostly known for their function in muscle sarcomeres. Although they may also play roles in neurons, they were excluded from further studies because of their large size, which makes molecular biology approaches challenging.

Next, the expression patterns of the identified genes were investigated using the Allen Brain Atlas (mouse brain). *MYO7B* was not found in the Allen Brain Atlas, and a literature search indicated that it is not expressed in the brain (Chen et al., 2001). *MYO15A* seemed to show very weak expression in the brain. Thus, these two myosins were excluded from further experiments. Attempts to clone *TRIO* constructs were unsuccessful and therefore the final study was carried out with five genes: *ACTN4, MYO9B, SWAP70, MYH10*, and *SRGAP3*. The mutations we investigated are all unique, one-allele *de novo* mutations leading to mixed expression of wild-type and mutated proteins. The selected genes also have other mutations; currently, the SFARI Gene database reports 3 variants for *ACTN4* (inheritance pattern unknown or *de novo*), 27 variants for *MYO9B* (both familial and *de novo*) and 4 variants for *SRGAP3* (all *de novo*). *SWAP70* and *MYH10* are not listed in the SFARI Gene database.

Alpha(α)-actinin-4 (*ACTN4*) is expressed in the hippocampus, cortex, and cerebellum in the brain (Kalinowska et al., 2015). The main function of α-actinins is to cross-link actin filaments into bundles (Otey and Carpen, 2004). Cross-linking actin filaments provides the rigidity and stability for filaments. In neurons, α-actinin-4 is enriched at excitatory synapses and co-localizes with group 1 metabotropic glutamate receptors (mGluRs) (Kalinowska et al., 2015). α-actinin-4 supports the transition of thin spines to mushroom spines and is required for the mGluR-induced dynamic remodeling of dendritic protrusions (Kalinowska et al., 2015).

Non-muscle myosin IIb (*MYH10*), or non-muscle myosin heavy chain IIb (NMMHCIIb) (referred to here as myosin IIb) is important for the normal development and function of dendritic spines (Zhang, 2005; Ryu et al., 2006; Rex et al., 2010; Hodges et al., 2011) and is re-located into dendritic spines during neuronal maturation (Ryu et al., 2006). Myosin IIb localizes to the base of mushroom spine heads (Korobova and Svitkina, 2010; Rubio et al., 2011) where it facilitates the stabilization of spines through actin cross-linking (Koskinen et al., 2014). Simultaneously, myosin IIb-induced contractility enhances the dynamics of actin filaments on the spine surface, thus facilitating the fast fine-tuning of spine shape (Koskinen et al., 2014). The *MYH10* gene is associated with various neurological diseases, such as schizophrenia and autism (Fromer et al., 2014).

The human myosin IXb (*MYO9B*) protein is mostly studied in cancer cell lines, where it localizes to sites of actin polymerization (van den Boom et al., 2007). However, myosin IXb is also expressed in the central nervous system. In cortical neurons, it controls RhoA activity, thereby regulating the growth and branching of dendritic processes (Long et al., 2013). Knockdown of myosin IXb in cultured cortical neurons or in the developing cortex results in decreased dendrite length and number (Long et al., 2013).

SWAP-70 (*SWAP70*) takes part in DNA recombination in the nucleus (Borggrefe et al., 1998) and regulates the actin cytoskeleton in the cytosol (Hilpelä et al., 2003; Chacón-Martínez et al., 2013). SWAP-70 binds the plasma membrane through the binding of its plecstrin homology domain to phosphoinositide PI(3,4)P2 (Hilpelä et al., 2003). SWAP-70 was identified as an interaction partner of myosin IXb in a two-hybrid screening (Hilpelä et al., 2003), but this putative interaction remains to be confirmed. Although SWAP-70 is expressed in the brain (Hilpelä et al., 2003), its function has not been studied in neurons.

SLIT-ROBO Rho GTPase-activating protein 3 (SrGAP3, Gene: *SRGAP3*) is ubiquitously expressed in the developing nervous system (Bacon et al., 2009, 2013). SrGAP3 supports the initiation of spines and inhibits the transition of thin spines to mushroom spines. Knocking out *SrGAP3* decreases the number of dendritic filopodia during early mouse development (Carlson et al., 2011).

The most commonly observed dendritic spine phenotype associated with ASD is an increased density of thin spines (Comery et al., 1997). Thus, our main hypothesis was that genetic mutations associated with ASD shift dendritic spine morphology from mushroom spines to thin spines. To test this hypothesis, we studied how ASD-associated single base-pair *de novo* mutations in five selected genes affect the localization and function of the encoded proteins, and whether these mutations change the proteins' overexpression effects on dendritic spine density and morphology in primary rat hippocampal neurons.

As the imbalance between excitation and inhibition has been suggested to have a central role in ASD (Rubenstein and Merzenich, 2003; Südhof, 2008; Uzunova et al., 2016; Lee et al., 2017), we analyzed the density and size of inhibitory synapses. Postmortem neuropathological studies of people with ASD have demonstrated a decreased density of GABA receptors in the cortex (Blatt and Fatemi, 2011). In mouse models, both upregulation and suppression in inhibitory synaptic transmission have been detected (Isshiki et al., 2014). Thus, we did not have clear expectations regarding the kind of changes we should see. Therefore, we hypothesized that there are alterations in the size and density of inhibitory synapses in neurons expressing proteins with ASD-linked mutations. We further hypothesized that the localization of inhibitory synapses in spines vs. dendritic shafts affects the efficiency or modality of inhibition. Thus, we also analyzed the ratio of inhibitory synapses in spines vs. dendritic shaft. None of the proteins studied was found to be part of the inhibitory synapse complex (Uezu et al., 2016) so we did not expect them to directly regulate inhibitory synapses. However, these proteins could affect inhibitory synapse dynamics and thus their localization, size and density through the regulation of actin dynamics (Wierenga, 2017).

## Materials and methods

### Plasmid construction

pEGFP-N1 and mCherry-C1 plasmids were purchased from Clontech Laboratories Inc. Wild-type mCherry-tagged α-actinin 4 (ACTN4) and Slit-Robo GAP3 (SRGAP3) were generated at the Genome Biology Unit cloning service (Biocenter Finland, University of Helsinki). Briefly, entry clones [clone-IDs: 100011237 (ACTN4) and 100069053 (SrGAP3)] from the human ORFeome collaboration library were transferred into mCherry-tagged mammalian expression destination vectors using standard LR Gateway cloning. mCherry-tagged SWAP-70, myosin IXb, and myosin IIb constructs were kind gifts from Martin Bähler (SWAP-70 and Myosin IXb, WestfalianWilhelms-University, Münster, Germany) and Alan Rick Horwitz (MHCIIb, University of Virginia, USA). Mutant constructs were generated through PCR-mediated site-directed mutagenesis (Zheng et al., 2004). Purified PCR products were treated for 2 h using DpnI (New England Biolabs) and 5 μl were used to transform competent DH5-α cells. Transformants were inoculated on LB plates containing appropriate antibiotics for selection and 5 colonies were selected for mini-prep. All wild-type and mutated constructs were verified by DNA sequencing. The primers used for each mutation are listed in Table 1. Mutagenized bases are listed in lower-case.

**Table 1:** The primers used for each mutation.

**Construct**	**Mutation**	**Primers**	
ACTN4	M554V	*forward*:	CAGGACgTGTTCATCGTCCATACCATCGAGGAG
		*reverse*:	GTATGGACGATGAACAcGTCCTGGAGGTCCTCC
MYO9B	K1872R	*forward*:	GATGCTCATCcgGGAACAGATGAGGAAATACAAGG
		*reverse*:	CTGTTCCcgGATGAGCATCTCCACACACG
SRGAP3	E469K	*forward*:	CTGGGCaAAGGGGAAAGAGCAGAATGCG
		*reverse*:	CTTTCCCCTTtGCCCAGGGTCTGCTTGAG
SWAP70	L544F	*forward*:	CCATCATGAAGGATTcATTCGACTGATAGAACCAGG
		*reverse*:	GTCGAATgAATCCTTCATGATGGGCCACTTTG
MYH10	Y265C	*forward*:	GATGTAACTGGCTgTATCGTTGGGGCCAACATTG
		*reverse*:	CCCAACGATAcAGCCAGTTACATCAAAGTTGATCCG

Plasmids were first tested in U2OS cells to confirm that they were expressed and localized as expected (data not shown).

### Neuronal cultures, transfections, immunofluorescence, and fixed sample preparation

Hippocampal neuronal cultures were prepared as described previously (Bertling et al., 2012). Animals were handled in accordance with Finnish laws and ethics under the EU directive 2010/63/EU (licenses: ESAVI-4943-04.10.07-2016 and GMO 3/S/12). Briefly, the hippocampi of embryonal day 17 Wistar rat fetuses of either sex were dissected and brains obtained. The meninges were then removed and hippocampi isolated. Cells were dissociated with 0.05% papain and mechanical trituration. The cells were plated on coverslips (diameter 13 mm) coated with 0.1 mg/ml Poly-L-Lysine (Sigma) at a density of 150,000 cells per coverslip and cultured in Neurobasal medium (Gibco) supplemented with B-27 (Invitrogen), L-glutamine (Invitrogen) and penicillin–streptomycin (Lonza). Transient transfections were performed, as described earlier (Hotulainen et al., 2009), on DIV13-14 using Lipofectamine 2000 (Invitrogen). The neurons were then fixed on DIV 15-16 with 4% PFA for 20 min, then washed 3 times with PBS. For inhibitory synapse labeling, the cells were then permeabilized with 0.2% TritonX-100 in PBS for 10 min and then blocked with 3% normal donkey serum and 0.5% BSA in PBS for 30 min. Antibodies were diluted separately in blocking solution at 1:500 for primary anti-gephyrin antibody (rabbit, Synaptic Systems) and 1:400 for secondary Alexa-488 (anti-rabbit, Molecular probes), and subsequently 25 μl was dropped onto a parafilm that was then covered by the coverslip. Each antibody was incubated for 1 h at room temperature. The coverslips were washed 3 times for 10 min with 0.2% BSA in PBS after each incubation, then mounted on microscope slides using Shandon Immu-Mount (Thermo Scientific).

### Confocal imaging and protein localization analysis

Imaging was performed on either a Zeiss LSM780 or LSM880 inverted confocal microscope. A 63× 1.4 NA oil immersion objective lens and Immersol 518F (Zeiss) immersion oil were used to image the fixed samples. For each neuron, a *z*-stack of 20–30 optical sections was obtained with 0.2–0.3 μm steps in the *z* axis and a pixel size of 0.066 × 0.066 μm for 1–2 dendritic segments. Image files were processed with Zeiss ZEN and Fiji software (Schindelin et al., 2012, 2015). Fiji software was used to calculate spine–to-dendrite intensity ratios. First, 3D image data was converted to 2D using Z-projection based on maximum intensity. To measure the ratio, the average intensity of fluorescence for the protein of interest was measured from a circular region of interest (ROI) in the spine head and compared to the average fluorescence intensity of the equal-sized ROI in the adjacent dendrite. The result was normalized to the intensity distribution of GFP in the same ROIs. For each neuron 9–10 spines were analyzed and the median value was then taken as representative for the cell. Therefore, for each group we analyzed at least 140 spines. Box plots were then created using a BoxPlotR web-tool (http://shiny.chemgrid.org/boxplotr/).

### Dendritic spine morphology and density analysis

For the analysis of spine density and morphology, tiff image files comprising *z*-stacks of 20–30 optical sections per dendritic segment were directly processed with NeuronStudio, a software package specifically designed for spine detection and analysis (Rodriguez et al., 2008). The voxel size of the images was 0.066 × 0.066 × 0.2–0.3 μm. The EGFP signal was used to analyze dendritic morphology. After modeling the dendrite surface, the spines were auto-detected and classified as “mushroom,” “thin,” or “stubby” by the software. The model was then corrected manually: the protrusions missed by the algorithm were added and classified, the false labeling of other structures as spines was removed. Protrusions with a length between 0.1 and 5 μm, and width between 0.1 and 3 μm were retained as spines. Measurements obtained by NeuronStudio were further processed in MATLAB R2015a to extract spine number, lengths, head widths, and the total length of dendritic segments, and summarized in a spreadsheet application (MS Excel).

### Density and size analysis of inhibitory synapses

Fiji software was used for the analysis of the density and size of inhibitory synapses. 3D images were opened in Fiji and used to create maximum intensity projections. The “straight line” tool was used to measure the length of the selected portion of the image. Lines were drawn, and the length was measured along the length of a dendrite. The data was then copied to an excel file and the sum of all the lines obtained gave the total length of the dendrite/s. The number of individual inhibitory synapses along the dendrite and the dendritic spines, indicated by bright green dots, was determined. Dots smaller than 0.1 μm were disregarded. The same “straight line” tool was used to measure the diameter of each synapse. The density, percentage of synapses on spines, and average diameter of synapses for inhibitory synapses was then calculated from the data obtained.

### Statistics

Statistical analysis was performed with the SPSS Statistics software package. To examine group differences, one-way ANOVA was used with Bonferroni *post-hoc* test when the compared groups' variances were equal and Games-Howell *post-hoc* test when the variances were unequal. To examine the difference in spine length, width, and width-to-length ratio distributions between the groups, we used the 2-sample Kolmogorov-Smirnov nonparametric test.

### Figures and molecular modeling

All structural figures were generated with PyMol software (The PyMOL Molecular Graphics System, Version 2.0 Schrödinger, LLC). Images were prepared for the publication using standard tools of Fiji software (Schindelin et al., 2012, 2015). The bar charts were created in MS Excel and the cumulative distribution curves in MATLAB R2015a. The final figure layouts were generated with Inkscape: Open Source Scalable Vector Graphics Editor.

### Availability of materials and data

All material and data (pictures, plasmids, analyses) are available upon request.

## Results

### α-Actinin-4 point mutation M554V alters localization and overexpression-induced dendritic spine phenotype

**Figure 1:**
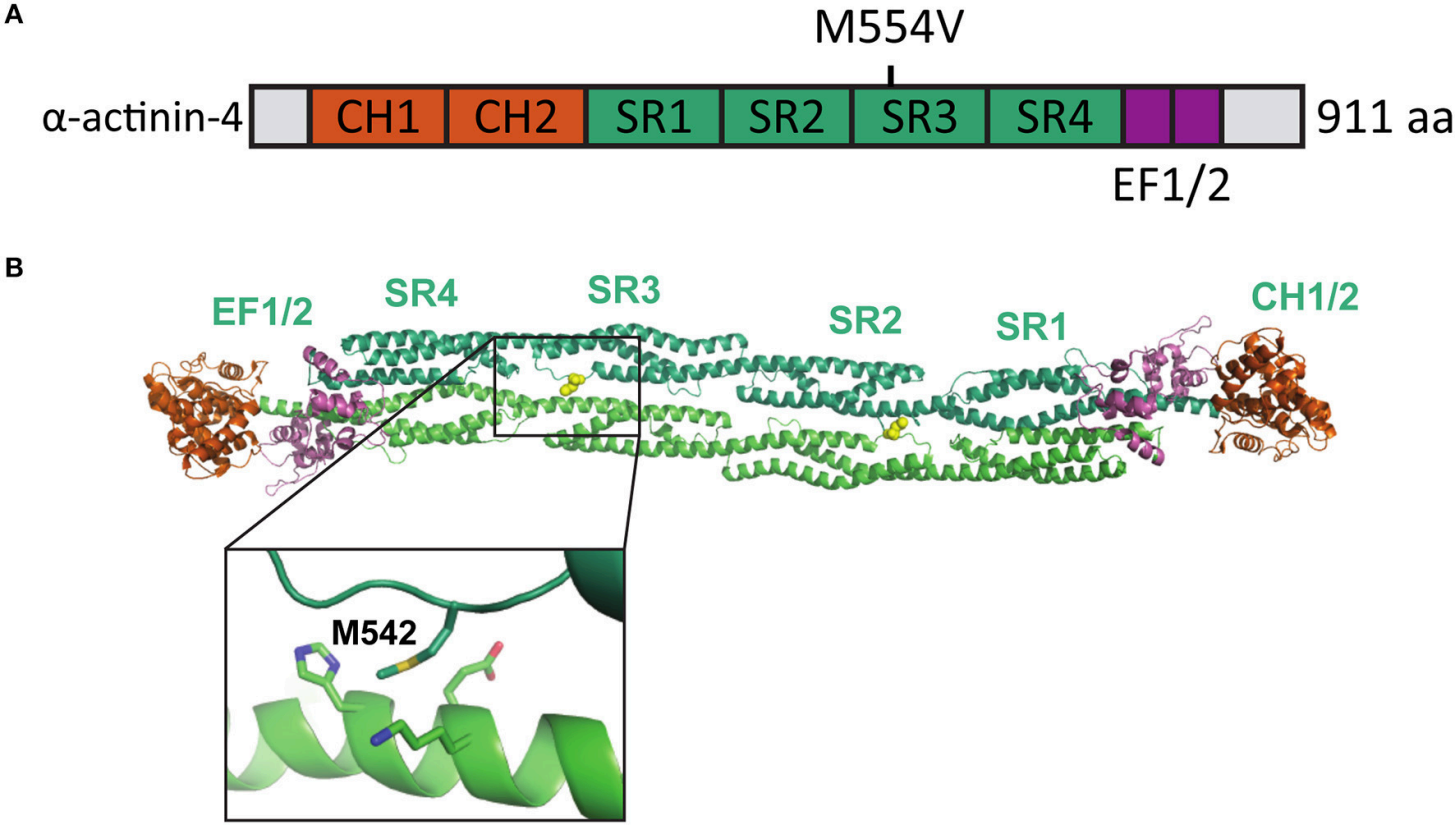
Location of the mutated amino acid in the α-actinin structure. **(A)** Primary structure of human α-actinin-4 with the M554V mutation indicated. **(B)** Structure of human α-actinin-2 (PDB ID 4D1E) with methionine corresponding to the α-actinin-4 M554 and surrounding residues highlighted (close-up).

Iossifov et al. (2012) identified a missense A to G change in the *ACTN4* gene in a child exhibiting ASD symptoms. This base change leads to an amino acid substitution [methionine (M) 554 to valine (V)] in the spectrin repeat-3 of the α-actinin-4 protein (Figure 1A). The formation of homodimers is required for efficient α-actinin actin filament cross-linking activity. As the M554V mutation localizes to the known binding interface of the α-actinin homodimer (Ylänne et al., 2001) (Figure 1B), we hypothesized that this mutation may prevent or interfere with the correct assembly of cellular actin filament bundles.

**Figure 2:**
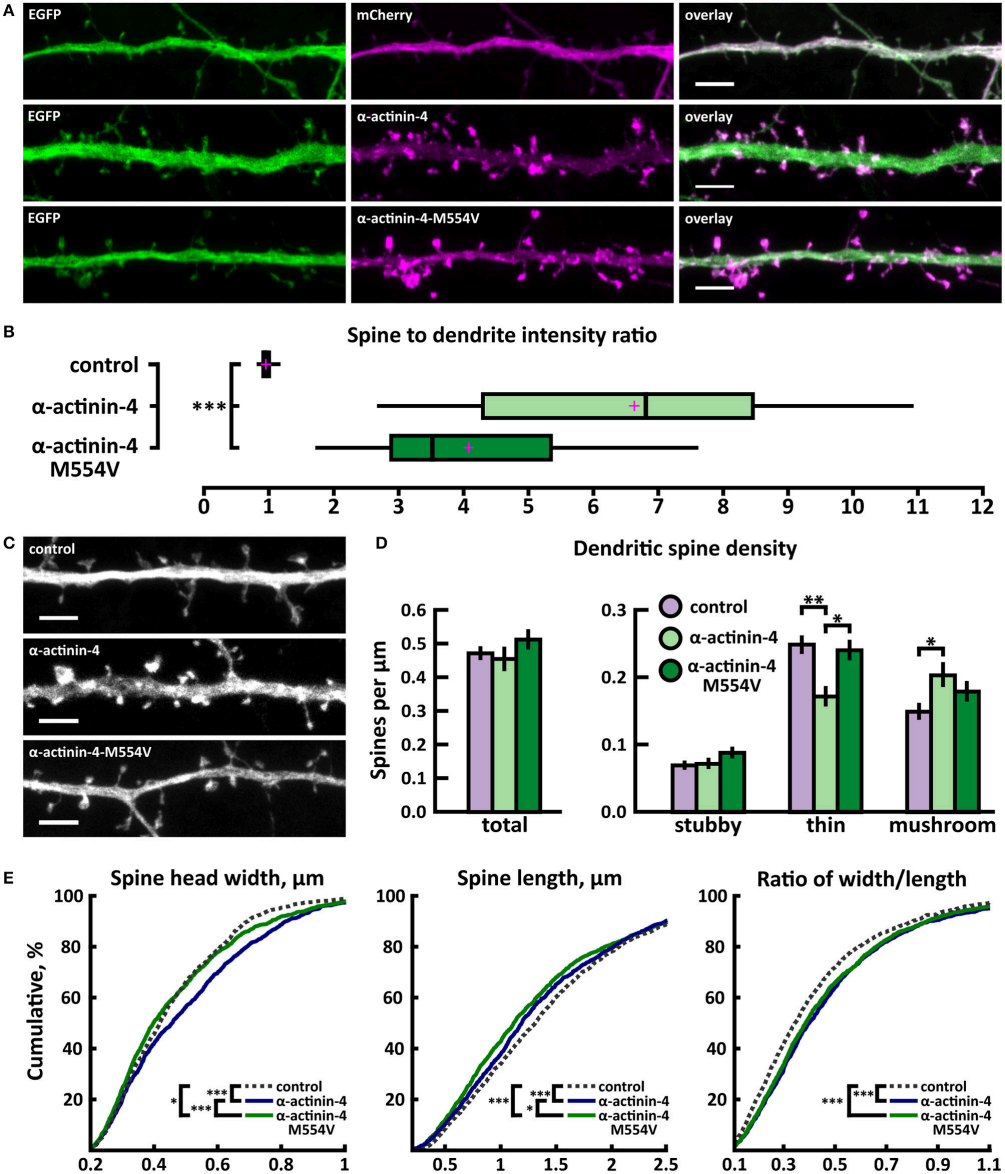
Characterization of protein localization, dendritic spine density and morphology in DIV14-15 hippocampal neurons expressing EGFP and mCherry (control), wild-type α-actinin-4-mCherry or mutated α-actinin-4-M554V-mCherry. **(A)** Representative maximum projections of confocal Z-stacks for EGFP, mCherry, and wild-type and mutated α-actinin-4 localization. Scale bars, 4 μm. **(B)** The quantification of protein localization revealed enrichment of wt α-actinin-4 to dendritic spines and the decrease in accumulation for α-actinin-4-M554V. Spine-to-dendrite fluorescence intensity ratios: control: 0.98 (*n* = 34 cells, 339 spines), α-actinin-4: 6.64 (*n* = 26 cells, 260 spines), α-actinin-4-M554V: 4.1 (*n* = 30 cells, 299 spines). Center lines show the medians; box limits indicate the 25th and 75th percentiles as determined by R software; whiskers extend 1.5 times the interquartile range from the 25th and 75th percentiles, outliers are represented by dots; crosses represent sample means. ***p < 0.001 as determined by one-way ANOVA test with Games-Howell *post-hoc* test. **(C)** Maximum projections of confocal Z-stacks of dendrites from neurons overexpressing EGFP along with either mCherry, α-actinin-4-mCherry or α-actinin-4-M554V-mCherry. Only EGFP channel is shown and was used to assess spine density and morphology. Scale bar, 4 μm. **(D)** Quantification of dendritic spine density calculated as number of spines per 1 μm of dendrite. The first left cluster of bars represents total spine density. Densities of thin, mushroom, stubby, and total spines were as follows: wt: thin = 0.25, mushroom = 0.15, stubby = 0.07, total = 0.48 spines/μm (*n* = 34 neurons, 1,937 spines, 4,091 μm dendrite); α-actinin-4: thin = 0.18, mushroom = 0.21, stubby = 0.08, total = 0.46 spines/μm (*n* = 26 neurons, 1,229 spines, 2,816 μm dendrite); α-actinin-4-M554V: thin = 0.24, mushroom = 0.18, stubby = 0.09, total = 0.51 spines/μm (*n* = 30 neurons, 1,750 spines, 3,573 μm dendrite). Data is pooled from 4 experiments and represented as mean ± SEM. *p < 0.05, **p < 0.01 as determined by one-way ANOVA test with Bonferroni *post-hoc* test. **(E)** Cumulative distributions of the width, length and the ratio of width to length of dendritic spines for neurons expressing either mCherry (control), α-actinin-4-mCherry or α-actinin-4-M554V-mCherry. Curves are a combination of data points each representing an individual spine. Matching tail regions of the curves are not shown. *p < 0.05, ***p < 0.001 as determined by pairwise two-sample Kolmogorov-Smirnov test.

To analyze the effects of M554V on α-actinin-4 subcellular localization, we transfected primary rat hippocampal neurons at days*-in-vitro* (DIV) 13 and analyzed the ratio of spine to dendritic localization of mCherry, human wild-type α-actinin-4 linked to mCherry, or mCherry-M554V-α-actinin-4 at DIV15. Fluorescence intensity was normalized to co-expressed EGFP (Figure 2A). Both α-actinin-4 constructs were expressed at similar levels in the cells analyzed. Wild-type α-actinin-4 was highly enriched in dendritic spines at a ratio of 6.64 ± 0.50 compared to diffuse GFP with a ratio of 0.98 ± 0.01 (Figures 2A,B, Table 2). M554V-α-actinin-4 also localized to dendritic spines—albeit to a much lesser extent than wild-type α-actinin-4 at a ratio of 4.10 ± 0.29 (Figures 2A,B).

**Table 2:** Result summary table.

**Comparison of wild-type (wt) protein phenotypes to control phenotype and mutant expression phenotype to wild-type phenotype**					
	α**-actinin-4**	**Myosin IIb**	**Myosin IXb**	**SWAP-70**	**SrGAP3**
**Localization to spines (ratio spine/shaft)**					
Ctrl vs. wt	0.98 vs. 6.64	0.93 vs. 1.73	0.92 vs. 1.65	0.98 vs. 1.15	0.99 vs. 1.32
Ctrl vs. mut	0.98 vs. 4.1	0.93 vs. 1.79	0.92 vs. 1.61	0.98 vs. 1.78	0.99 vs. 1.42
wt vs. mut	6.64 vs. 4.1	1.73 vs. 1.79	1.65 vs. 1.61	1.15 vs. 1.78	1.32 vs. 1.42
**Total spine density (spines/**μ**m)**					
Ctrl vs. wt	0.48 vs. 0.46	0.55 vs. 0.57	0.48 vs. 0.44	0.39 vs. 0.46	0.45 vs. 0.54
Ctrl vs. mut	0.48 vs. 0.51	0.55 vs. 0.61	0.48 vs. 0.49	0.39 vs. 0.54	0.45 vs. 0.54
wt vs. mut	0.46 vs. 0.51	0.57 vs. 0.61	0.44 vs. 0.49	0.46 vs. 0.54	0.54 vs. 0.54
**Thin spine density (spines/**μ**m)**					
Ctrl vs. wt	0.25 vs. 0.18	0.25 vs. 0.27	0.24 vs. 0.24	0.19 vs. 0.25	0.23 vs. 0.29
Ctrl vs. mut	0.25 vs. 0.24	0.25 vs. 0.27	0.24 vs. 0.27	0.19 vs. 0.31	0.23 vs. 0.28
wt vs. mut	0.18 vs. 0.24	0.27 vs. 0.27	0.24 vs. 0.27	0.25 vs. 0.31	0.29 vs. 0.28
**Mushroom spine density (spines/**μ**m)**					
Ctrl vs. wt	0.15 vs. 0.21	0.20 vs. 0.21	0.18 vs. 0.15	0.16 vs. 0.18	0.16 vs. 0.18
Ctrl vs. mut	0.15 vs. 0.18	0.20 vs. 0.22	0.18 vs. 0.15	0.16 vs. 0.18	0.16 vs. 0.19
wt vs. mut	0.21 vs. 0.18	0.21 vs. 0.22	0.15 vs. 0.15	0.18 vs. 0.18	0.18 vs. 0.19
**Total spine head size (**μ**m)**					
Ctrl vs. wt	0.46 vs. 0.46	0.49 vs. 0.43	0.46 vs. 0.42	0.44 vs. 0.44	0.44 vs. 0.45
Ctrl vs. mut	0.46 vs. 0.46	0.49 vs. 0.49	0.46 vs. 0.43	0.44 vs. 0.40	0.44 vs. 0.45
wt vs. mut	0.46 vs. 0.46	0.43 vs. 0.49	0.42 vs. 0.43	0.44 vs. 0.40	0.45 vs. 0.45
**Inhibitory synapse density (synapses/**μ**m)**					
Ctrl vs. wt	0.40 vs. 0.31	0.37 vs. 0.45	0.37 vs. 0.37	0.38 vs. 0.38	0.37 vs. 0.41
Ctrl vs. mut	0.40 vs. 0.25	0.37 vs. 0.39	0.37 vs. 0.40	0.38 vs. 0.28	0.37 vs. 0.51
wt vs. mut	0.31 vs. 0.25	0.45 vs. 0.39	0.37 vs. 0.40	0.38 vs. 0.28	0.41 vs. 0.51
**Inhibitory synapse size (**μ**m)**					
Ctrl vs. wt	0.43 vs. 0.44	0.44 vs. 0.46	0.44 vs. 0.48	0.42 vs. 0.38	0.44 vs. 0.38
Ctrl vs. mut	0.43 vs. 0.44	0.44 vs. 0.42	0.44 vs. 0.44	0.42 vs. 0.40	0.44 vs. 0.39
wt vs. mut	0.44 vs. 0.44	0.46 vs. 0.42	0.48 vs. 0.44	0.38 vs. 0.40	0.38 vs. 0.39
**Proportion of inhibitory synapses in spines vs. shaft**					
Ctrl vs. wt	0.38 vs. 0.37	0.33 vs. 0.43	0.33 vs. 0.41	0.34 vs. 0.37	0.33 vs. 0.33
Ctrl vs. mut	0.38 vs. 0.31	0.33 vs. 0.34	0.33 vs. 0.39	0.34 vs. 0.34	0.33 vs. 0.42
wt vs. mut	0.37 vs. 0.31	0.43 vs. 0.34	0.41 vs. 0.39	0.37 vs. 0.34	0.33 vs. 0.42
Main changes for wt vs. control	Decreased thin spine density and increased mushroom spine density	Increased proportion of inhibitory synapses in spines	Increased proportion of inhibitory synapses in spines	No significant changes in spines or inhibitory synapses	Reduced inhibitory synapse size
Main changes for mutant vs. wt	Reduced localization to spines and increased thin spines density	Reduced size of inhibitory synapses and reduced proportion of inhibitory synapses in spines	No significant changes	Enhanced spine localization, reduced spine head size and reduced inhibitory synapse density	Increased localization to spines, increased proportion of inhibitory synapses in spines

*Values obtained from wild-type (ctrl vs. wt) or mutated protein (ctrl vs. mut) expressing cells are compared to control cells, and values obtained from cells expressing wild-type proteins are compared to values of mutant protein expressing cells (wt vs. mut). A statistically significant (p < 0.05) increase is highlighted with green and decrease with blue*.

To assess possible changes in dendritic spines, we analyzed dendritic spine morphology and density in neurons co-transfected with EGFP and wild-type or mutated α-actinin-4-mCherry. Dendritic spine density and morphology were analyzed using the NeuronStudio software (Rodriguez et al., 2008) using EGFP intensity. The overexpression of wild-type α-actinin-4 resulted in an increase in mushroom spine density, while mutated α-actinin-4 failed to induce such an increase when compared to control cells expressing mCherry (Figures 2C,D). Thin spine density was decreased in wild-type α-actinin-4 expressing cells compared to control cells, whereas in mutant α-actinin-4 expressing cells the thin spine density was significantly increased compared to wild-type expressing cells (Figures 2C,D, Table 2). The cumulative distributions of spine head width, length, and width-to-length ratios showed some significant differences. Wild-type—but not mutant—overexpressing cells had a higher proportion of spines with wider heads in the range of 0.2–0.8 μm, whereas both wild-type and mutant overexpressing cells had slightly higher proportions of shorter spines in the range of 0.5–2.5 μm (Figure 2E). From the distributions of width-to-length ratios of spines we can see that in cells overexpressing wild-type or mutant α-actinin-4, more spines possessed mature morphology (i.e., wide head, short neck) compared to controls. This suggests that changes in spine widths and lengths occur proportionally in the same spines. Taken together, these results show that mutated α-actinin-4 is less concentrated in dendritic spines and fails to mimic the effect of the wild-type protein on dendritic spine morphology.

**Figure 3:**
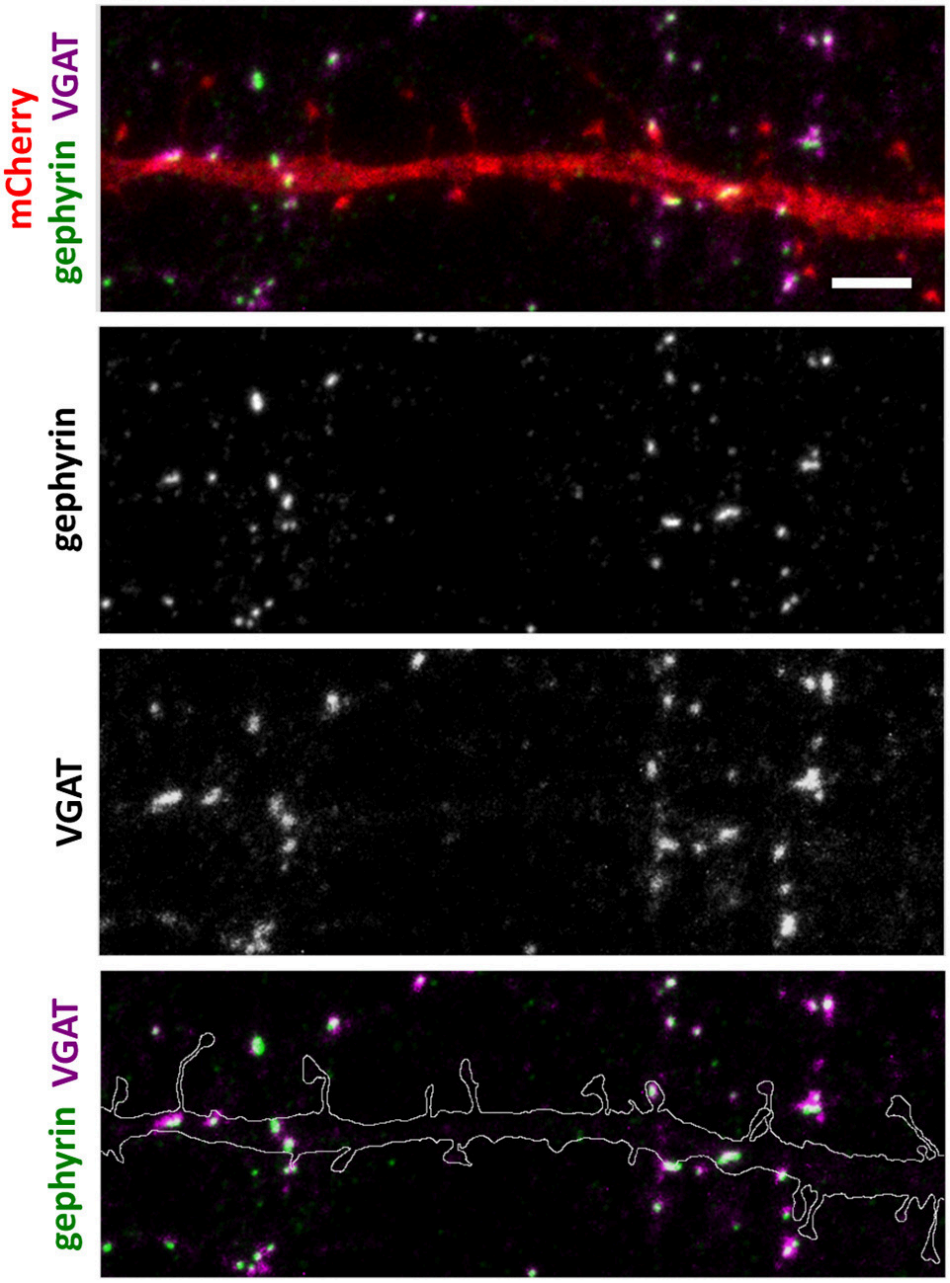
Inhibitory synapse localization in a DIV15 hippocampal neuron expressing mCherry and stained with postsynaptic anti-gephyrin (Alexa-488) and presynaptic anti-VGAT (Alexa-594) antibodies. In overlay, mCherry is shown in red, anti-gephyrin antibody staining in green, and anti-VGAT antibody staining in magenta. The line indicates the contour of dendrites (from mCherry). Scale bar, 5 μm.

**Figure 4:**
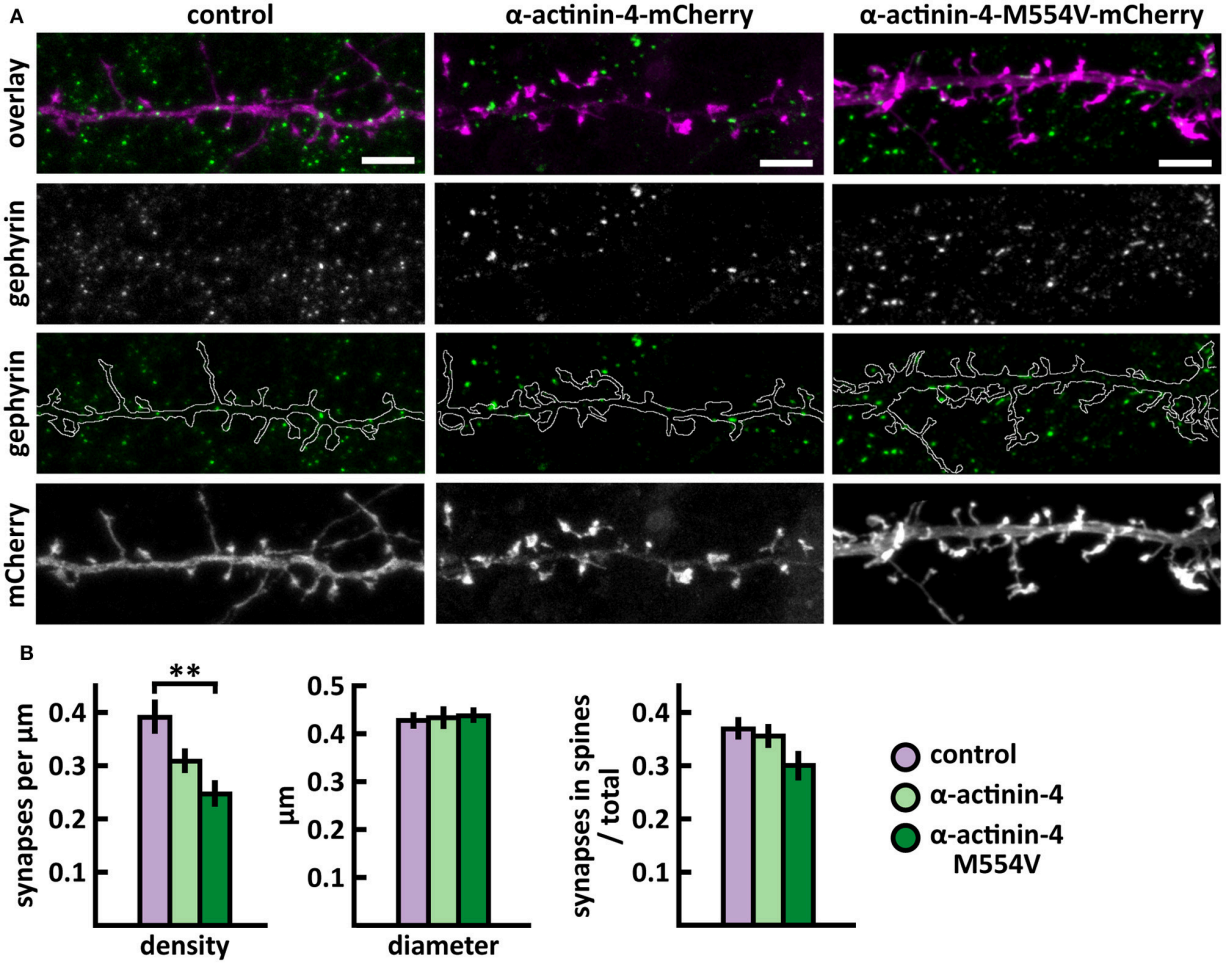
Characterization of the inhibitory synapses in DIV15 hippocampal neurons expressing either mCherry (control), wild-type α-actinin-4-mCherry or α-actinin-4-M554V-mCherry and stained with anti-gephyrin antibody. **(A)** In overlay, mCherry is shown in magenta and anti-gephyrin antibody staining in green. The line indicates the contour of dendrites (from mCherry). Scale bars, 5 μm. **(B)** Quantification of inhibitory synapse properties. From left to right: (1) Mean density of synapses calculated as the number of gephyrin puncta per μm of the dendrite: control = 0.40; α-actinin-4 = 0.31; α-actinin-4-M554V = 0.25. (2) Mean diameter of individual gephyrin puncta in μm: control = 0.43; α-actinin-4 = 0.44; α-actinin-4-M554V = 0.44. (3) Proportion of the gephyrin puncta located on dendritic spines as a fraction of the total number of puncta: control = 0.38; α-actinin-4 = 0.37; α-actinin-4-M554V = 0.31. Data in **(B)** represent n(control) = 19 cells, n(α-actinin-4) = 21 cells, n(α-actinin-4-M554V) = 20 cells, pooled from 3 experiments. ^**^p < 0.01 as determined by one-way ANOVA with Bonferroni's *post-hoc* test. Data is represented as mean ± SEM.

Next, we analyzed the density and size of inhibitory synapses, as well as the proportion of inhibitory synapses in spines, by visualizing inhibitory synapses using gephyrin antibody staining. To check the specificity of gephyrin staining, we co-stained cells with the pre-synaptic VGAT antibody (Figure 3). This co-staining showed co-localization between gephyrin puncta and the presynaptic VGAT marker, ruling out the possibility of unspecific gephyrin staining (Figure 3). Analysis of gephyrin puncta revealed that the overexpression of wild-type α-actinin-4 reduced the density of inhibitory synapses (Figure 4, Table 2). However, the change varied between experiments and, therefore, this result did not reach statistical significance. Mutated α-actinin-4 enhanced this effect of wild-type protein and the difference between mutant α-actinin-4 and control cells was significant (Figure 4B). The changes in synapse size or the proportion of synapses on dendritic spines were not significant (Figure 4B).

In summary, we found that M554V point mutation in α-actinin-4 reduces the localization of α-actinin-4 to dendritic spines. The expression of α-actinin-4-M554V in hippocampal neurons resulted in an increased thin spine density compared to the expression of wild-type α-actinin-4. The mutation in α-actinin-4 enhanced the effect of wild-type protein in decreasing the inhibitory synapse density, and the difference in density between control and mutant α-actinin-4 expressing neurons was significant. These results suggest that the ASD-associated M554V mutation leads to a loss-of–function or reduced-function effect in α-actinin-4 in the regulation of dendritic spines. However, this mutation enhances the effect of wild-type α-actinin-4 in inhibitory synapses (Table 2).

### Myosin IIb mutation Y265C expression reduces inhibitory synapse size and the proportion of inhibitory synapses in spines

**Figure 5:**
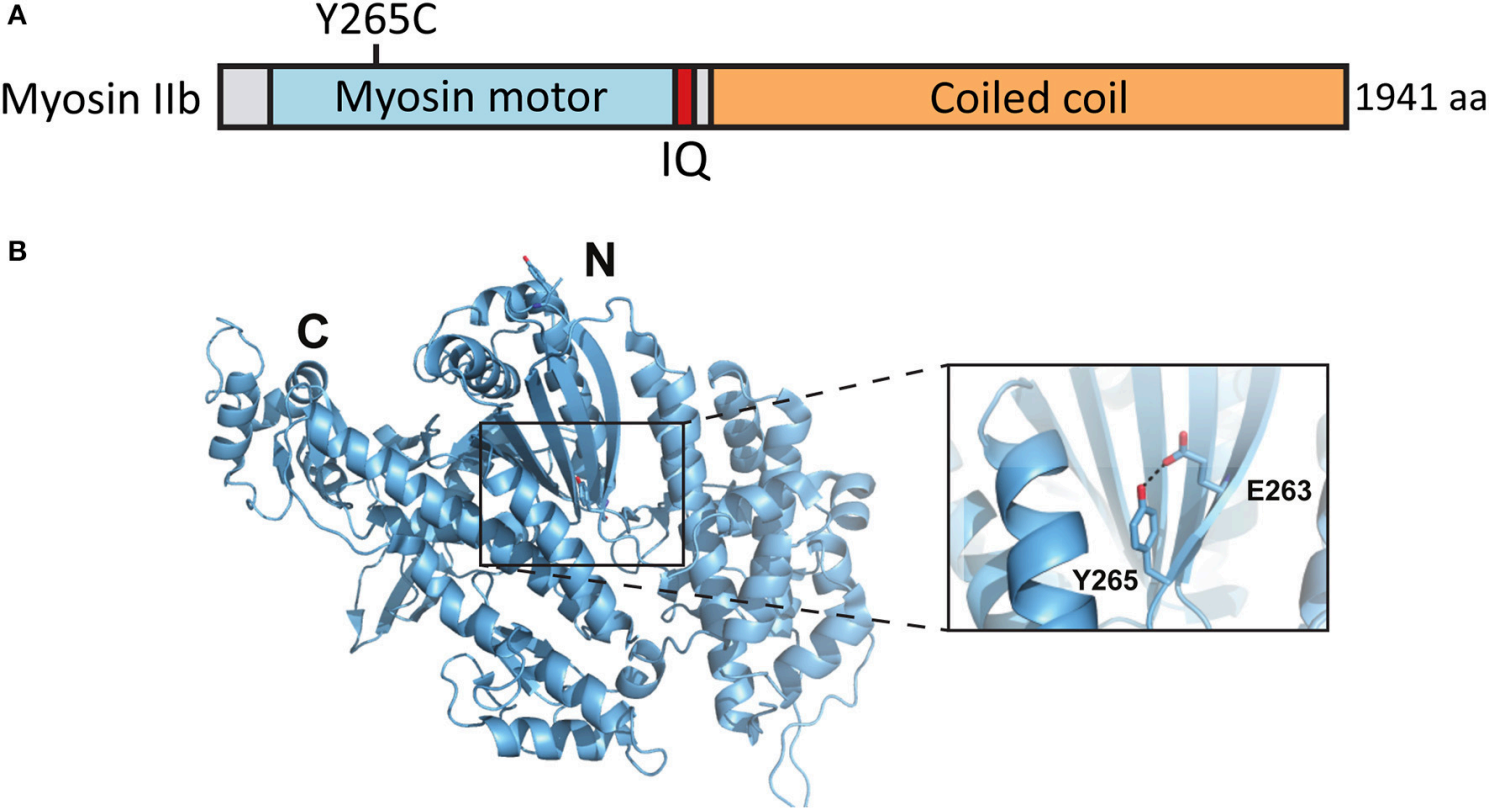
Location of the mutated amino acid in the myosin IIb structure. **(A)** Primary structure of human myosin IIb with the Y265C mutation indicated. **(B)** Structure of human myosin IIb (PDB ID 4PD3) with residue Y265 and surrounding residues highlighted (close-up).

A *de novo* missense mutation T to C in the *MYH10* gene was found by whole exome sequencing of parent–child trios exhibiting sporadic ASD (O'Roak et al., 2012) (Figures 5A,B). This base change leads to the substitution of tyrosine-265 (Y265) to cysteine (C) in the myosin IIb protein. Y265 is located in the center of the myosin IIb motor domain and participates in a potentially stabilizing hydrogen bond interaction with the neighboring glutamate-263. These residues are distal from the motor domain ATP binding site but could contribute to the ATPase activity by affecting the stability and overall folding of this domain (Figures 5A,B).

**Figure 6:**
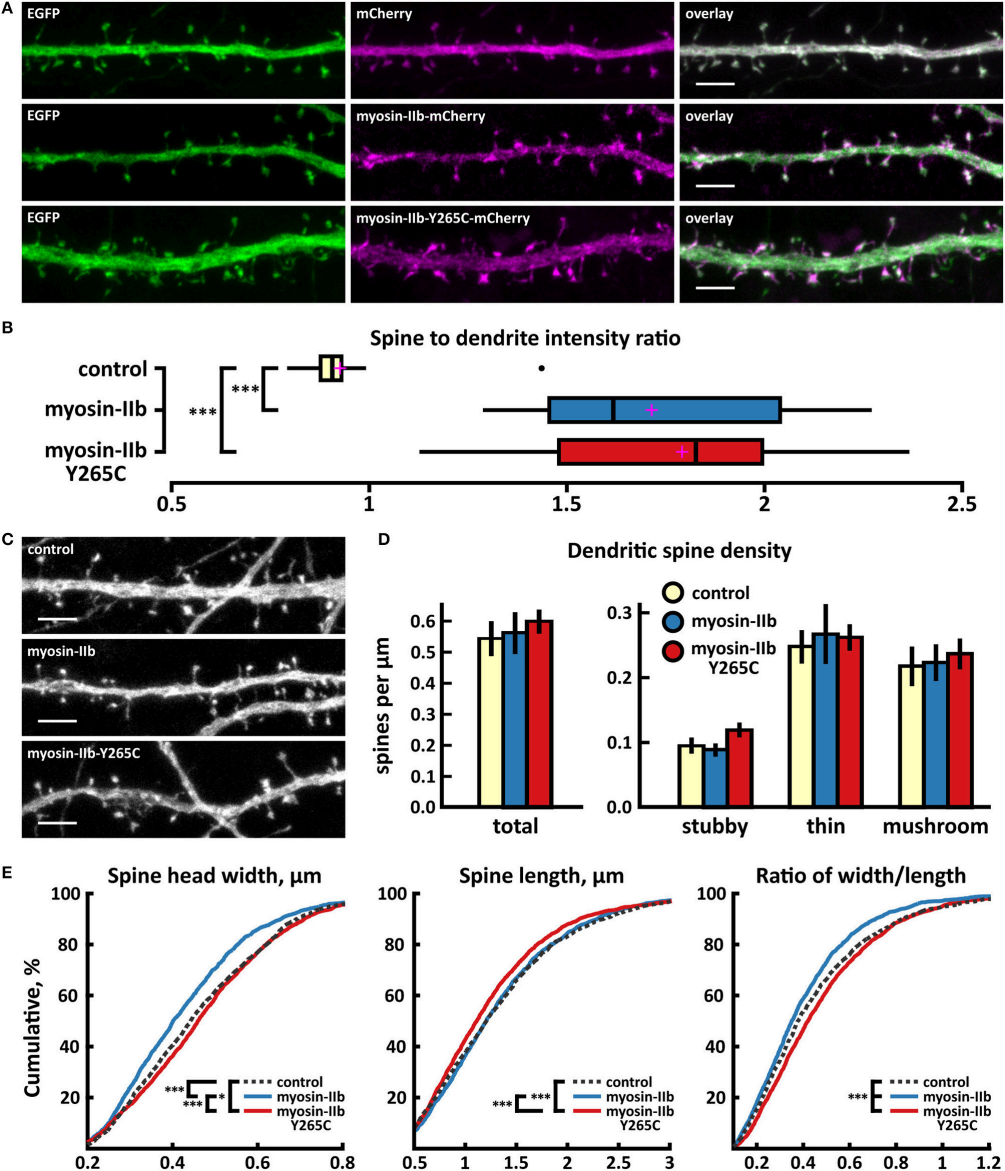
Characterization of protein localization, dendritic spine density and morphology in DIV14-15 hippocampal neurons expressing EGFP and mCherry (control), myosin-IIb-mCherry or mutated myosin-IIb-Y265C-mCherry. **(A)** Representative maximum projections of confocal Z-stacks for EGFP, mCherry, and wild-type and mutated myosin-IIb localization. Scale bars 4 μm. **(B)** The quantification of protein localization revealed similar enrichment for both wt and mutated myosin-IIb to dendritic spines. Spine-to-dendrite fluorescence intensity ratios: control: 0.93 (*n* = 17 cells, 170 spines), myosin-IIb: 1.73 (*n* = 17 cells, 170 spines), myosin-IIb-Y265C 1.79 (*n* = 18 cells, 180 spines). Center lines show the medians; box limits indicate the 25th and 75th percentiles as determined by R software; whiskers extend 1.5 times the interquartile range from the 25th and 75th percentiles, outliers are represented by dots; crosses represent sample means. ***p < 0.001 as determined by one-way ANOVA test with Games-Howell *post-hoc* test. **(C)** Maximum projections of confocal Z-stacks of dendrites from neurons overexpressing EGFP along with either mCherry, myosin-IIb-mCherry, or myosin-IIb-Y265C-mCherry. Only EGFP channel is shown and was used to assess spine density and morphology. Scale bar, 4 μm. **(D)** Quantification of dendritic spine density calculated as number of spines per 1 μm of dendrite. The first left cluster of bars represents total spine density. Densities of thin, mushroom, stubby, and total spines were as follows: wt: thin = 0.25, mushroom = 0.2, stubby = 0.1, total = 0.55 spines/μm (*n* = 17 neurons, 1,825 spines, 3,354 μm dendrite); myosin-IIb: thin = 0.27, mushroom = 0.2, stubby = 0.09, total = 0.57 spines/μm (*n* = 17 neurons, 1,718 spines, 3,459 μm dendrite); myosin-IIb-Y265C: thin = 0.27, mushroom = 0.22, stubby = 0.12, total = 0.61 spines/μm (*n* = 18 neurons, 2,253 spines, 3,877 μm dendrite). Data is pooled from 2 experiments and represented as mean ±SEM. Significant differences were not detected by one-way ANOVA test with Games-Howell or Bonferroni *post-hoc* test. **(E)** Cumulative distributions of the width, length, and the ratio of width to length of dendritic spines for neurons expressing either mCherry (control), myosin-IIb-mCherry or myosin-IIb-Y265C-mCherry. Curves are a combination of data points each representing an individual spine. Matching tail regions of the curves are not shown. *p < 0.05, ***p < 0.001 as determined by pairwise two-sample Kolmogorov-Smirnov test.

As expected from earlier work (Korobova and Svitkina, 2010; Rubio et al., 2011), wild-type myosin IIb localized to the base of the spine head, showing enrichment in spines vs. dendrites (Figures 6A,B). The mutated myosin IIb exhibited similar localization (Figures 6A,B). Spine analysis did not reveal any differences in spine density between control, myosin IIb wild-type, and myosin IIb-Y265C (Figures 6C,D). Cumulative distribution analysis showed slight changes in spine morphology (Figure 6E). Neurons overexpressing wild-type myosin IIb exhibited a smaller proportion of wider spines compared to controls and neurons overexpressing myosin-IIb-Y265C. Mutant-expressing neurons showed an increased fraction of wider spines and a decreased proportion of long spines compared to wild-type myosin IIb-expressing neurons. In summary, only minor changes in dendritic spines were observed upon myosin IIb-Y265C over-expression.

**Figure 7:**
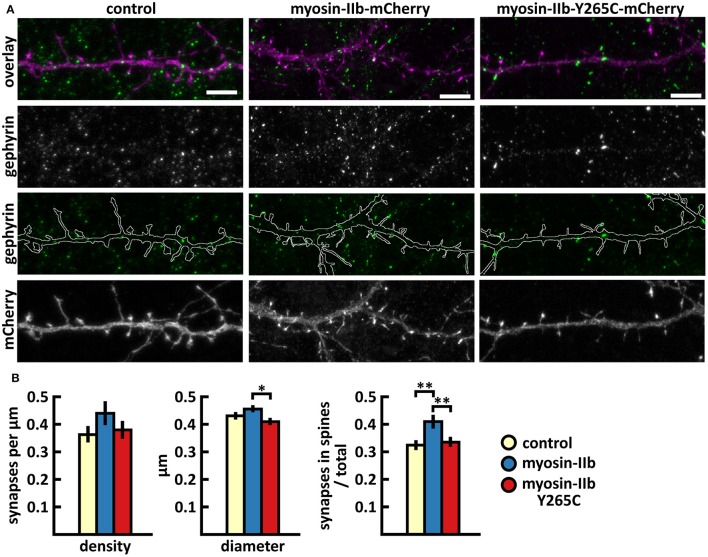
Characterization of the inhibitory synapses in DIV15 hippocampal neurons expressing either mCherry (control), wild-type myosin IIb-mCherry or myosin IIb-Y265C-mCherry and stained with anti-gephyrin antibody. **(A)** In overlay, mCherry is shown in magenta and anti-gephyrin antibody staining in green. The line indicates the contour of dendrites (from mCherry). Scale bars, 5 μm. **(B)** Quantification of inhibitory synapse properties. From left to right: (1) Mean density of synapses calculated as the number of gephyrin puncta per μm of the dendrite: control = 0.37; myosin IIb = 0.45; myosin IIb-Y265C = 0.39. (2) Mean diameter of individual gephyrin puncta in μm: control = 0.44; myosin IIb = 0.46; myosin IIb-Y265C = 0.42 3. Proportion of the gephyrin puncta located on dendritic spines as a fraction of the total number of puncta: control = 0.33; myosin IIb = 0.43; myosin IIb-Y265C = 0.34. Data in **(B)** represent n(control) = 17 cells, n(myosin IIb) = 20 cells, n(myosin IIb-Y265C) = 19 cells, pooled from 3 experiments. *p < 0.05 and ^**^p < 0.01 as determined by one-way ANOVA with Bonferroni's *post-hoc* test. Data is represented as mean ±SEM.

The analysis of inhibitory synapses showed that wild-type myosin IIb expression increased the proportion of inhibitory synapses in spines compared to control cells (Figures 7A,B, Table 2). In contrast, mutant-expressing cells had fewer inhibitory synapses located on dendritic spines compared to wild-type overexpressing neurons (Figures 7A,B, Table 2). Moreover, the size of inhibitory synapses was smaller in neurons overexpressing myosin IIb-Y265C compared to neurons expressing wild-type myosin IIb.

In summary, the Y265C-mutation induced slight changes in the morphology and size of dendritic spines compared to neurons expressing wild-type myosin IIb. The expression of the mutant reduced the size of inhibitory synapses and the proportion of inhibitory synapses located on spines, compared to wild-type expression.

### Myosin IXB increases the proportion of inhibitory synapses in spines

**Figure 8:**
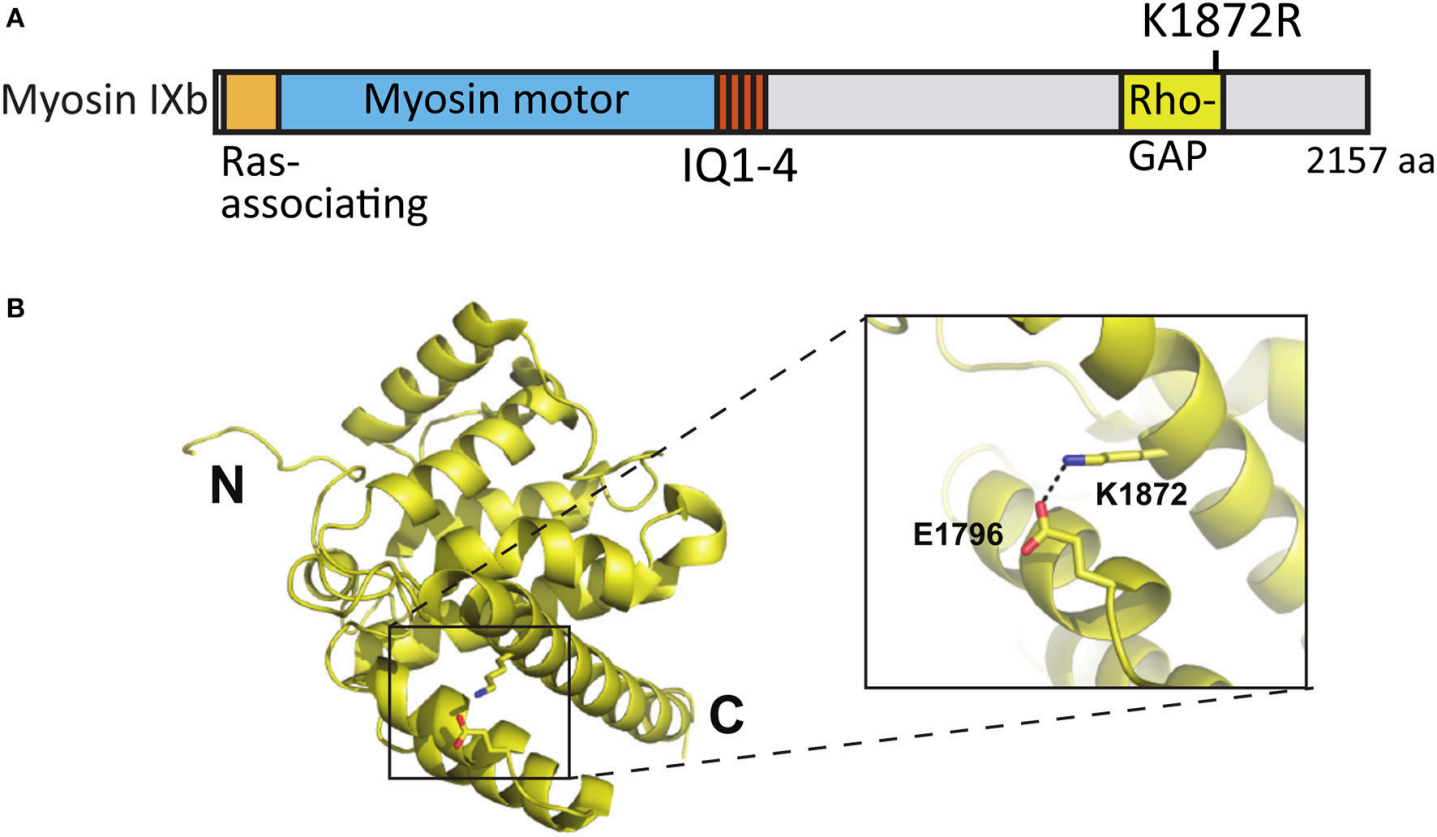
Location of the mutated amino acid in the myosin IXb structure. **(A)** Primary structure of human myosin IXb with the K1872R mutation indicated. **(B)** Structure of human myosin IXb (PDB ID 5C5S) with residue K1872 and surrounding residues highlighted (close-up).

Whole exome sequencing applied to families with an ASD child revealed A/G *de novo* missense mutations in the *MYO9B* gene leading to an amino acid change [lysine (K) 1872 to arginine (R)] in the RhoGAP domain of the myosin IXb protein (Iossifov et al., 2014) (Figure 8A). K1872 is involved in a stabilizing salt-bridge interaction with E1796 within the RhoGAP domain. However, it is not likely that the conservative lysine to arginine mutation would result in significant domain destabilization or functional change (Figures 8A,B).

**Figure 9:**
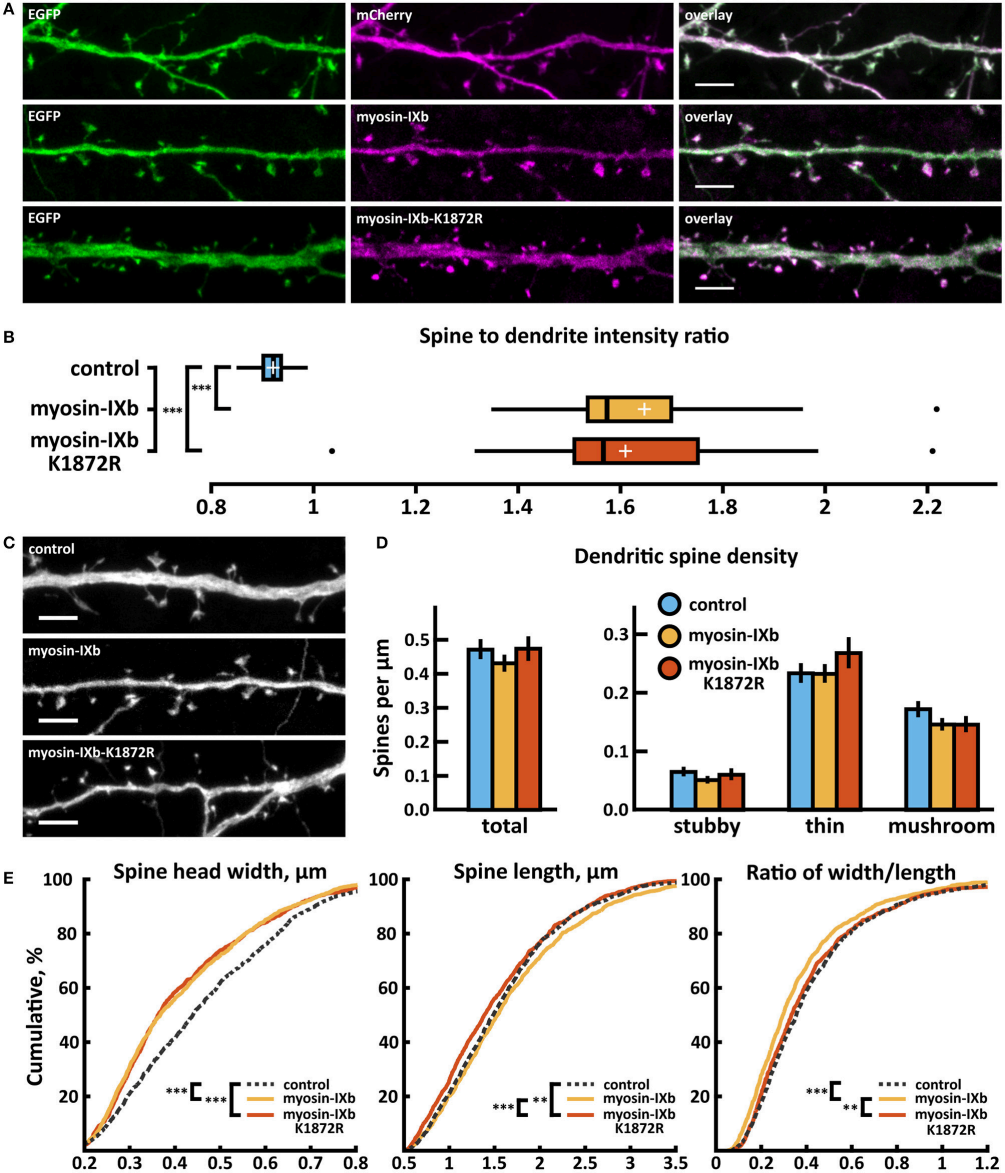
Characterization of protein localization, dendritic spine density and morphology in DIV14-15 hippocampal neurons expressing EGFP and mCherry (control), wild-type myosin-IXb-mCherry or mutated myosin-IXb-K1872R-mCherry. **(A)** Representative maximum projections of confocal Z-stacks for EGFP, mCherry, and wild-type and mutated myosin IXb localization. Scale bars, 4 μm. **(B)** The quantification of protein localization revealed revealed similar enrichment for both wt and mutated myosin-IXb to dendritic spines. Spine-to-dendrite fluorescence intensity ratios: control: 0.92 (*n* = 21 cells, 210 spines), myosin-IXb: 1.65 (*n* = 18 cells, 180 spines), myosin-IXb-K1872R: 1.61 (*n* = 14 cells, 140 spines). Center lines show the medians; box limits indicate the 25th and 75th percentiles as determined by R software; whiskers extend 1.5 times the interquartile range from the 25th and 75th percentiles, outliers are represented by dots; crosses represent sample means. ***p < 0.001 as determined by one-way ANOVA test with Games-Howell *post-hoc* test. **(C)** Maximum projections of confocal Z-stacks of dendrites from neurons overexpressing EGFP along with either mCherry, myosin-IXb-mCherry or myosin-IXb-K1872R-mCherry. Only EGFP channel is shown and was used to assess spine density and morphology. Scale bar, 4 μm. **(D)** Quantification of dendritic spine density calculated as number of spines per 1 μm of dendrite. The first left cluster of bars represents total spine density. Densities of thin, mushroom, stubby, and total spines were as follows: wt: thin = 0.24, mushroom = 0.18, stubby = 0.07, total = 0.48 spines/μm (*n* = 21 neurons, 1,567 spines, 3,321 μm dendrite); myosin-IXb: thin = 0.24, mushroom = 0.15, stubby = 0.06, total = 0.44 spines/μm (*n* = 18 neurons, 1,059 spines, 2,475 μm dendrite); myosin-IXb-K1872R: thin = 0.27, mushroom = 0.15, stubby = 0.06, total = 0.49 spines/μm (*n* = 14 neurons, 1,006 spines, 2,120 μm dendrite). Data is pooled from 3 experiments and represented as mean ±SEM. Significant differences were not detected by one-way ANOVA test with Bonferroni *post-hoc* test. **(E)** Cumulative distributions of the width, length, and the ratio of width to length of dendritic spines for neurons expressing either mCherry (control), myosin-IXb-mCherry or myosin-IXb-K1872R-mCherry. Curves are a combination of data points each representing an individual spine. Matching tail regions of the curves are not shown. **p < 0.01, ***p < 0.001 as determined by pairwise two-sample Kolmogorov-Smirnov test.

Over-expression of wild-type and myosin IXb-K1872R-mCherry constructs revealed enrichment in spine heads 1.6 times that of mCherry (Figures 9A,B). Spine analysis revealed no significant changes in spine density in neurons expressing either wild-type or myosin IXb-K1872R-mCherry constructs (Figures 9C,D, Table 2). Thin spine density was slightly increased in neurons expressing mutated myosin IXb, but this change did not reach statistical significance. Examination of the cumulative distribution of spine morphological aspects such as width, length, and width-to-length ratio showed only minor differences (Figure 9E). Neurons expressing wild-type or myosin IXb-K1872R had a marginally higher proportion of thinner spines in the range of 0.3–0.7 μm. The lengths and width-to-length ratios of spines had very comparable distributions for all three groups (Figure 9E).

**Figure 10:**
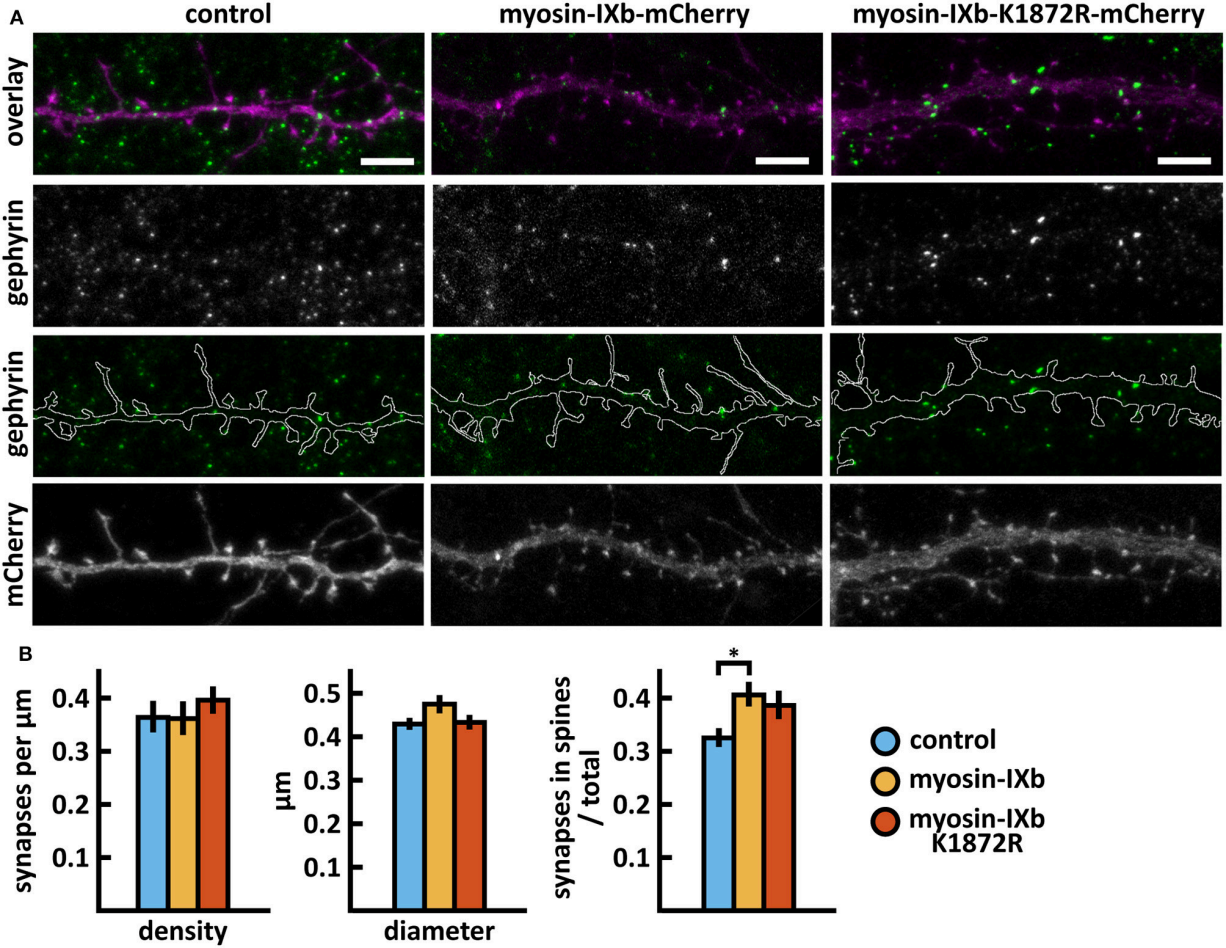
Characterization of the inhibitory synapses in DIV15 hippocampal neurons expressing either mCherry (control), wild-type myosin IXb-mCherry or myosin IXb-K1872R-mCherry and stained with anti-gephyrin antibody. **(A)** In overlay, mCherry is shown in magenta and anti-gephyrin antibody staining in green. The line indicates the contour of dendrites (from mCherry). Scale bars, 5 μm. **(B)** Quantification of inhibitory synapse properties. From left to right: (1) Mean density of synapses calculated as the number of gephyrin puncta per μm of the dendrite: control = 0.37; myosin IXb = 0.37; myosin IXb-K1872R = 0.40. (2) Mean diameter of individual gephyrin puncta in μm: control = 0.44; myosin IXb = 0.48; myosin IXb-K1872R = 0.44 3. Proportion of the gephyrin puncta located on dendritic spines as a fraction of the total number of puncta: control = 0.33; myosin IXb = 0.41; myosin IXb-K1872R = 0.39. Data in **(B)** represent n(control) = 17 cells, n(myosin IXb) = 24 cells, n(myosin IXb-K1872R) = 24 cells, pooled from 2 experiments. *p < 0.05 as determined by one-way ANOVA with Bonferroni's *post-hoc* test. Data is represented as mean ± SEM.

Wild-type myosin IXb expression increased the proportion of inhibitory synapses compared to control cells (Figures 10A,B, Table 2). The K1872R mutation did not cause any significant changes in inhibitory synapses when compared to wild-type expression.

### SWAP-70 mutant L544F expression enhances localization to spines and reduces the spine width and inhibitory synapse density

**Figure 11:**
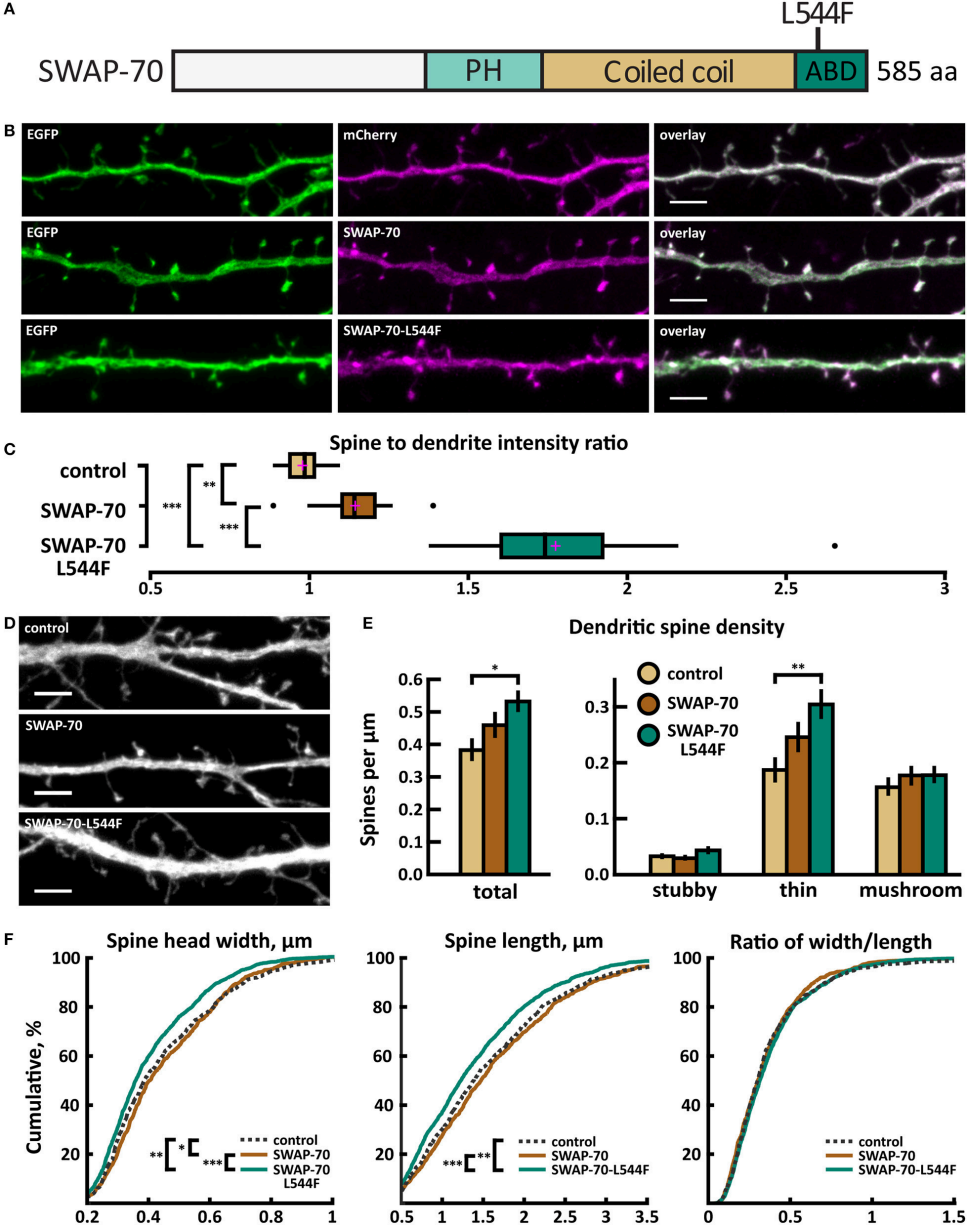
Characterization of protein localization, dendritic spine density and morphology in DIV14-15 hippocampal neurons expressing EGFP and mCherry (control), wild-type SWAP-70-mCherry or mutated SWAP-70-L544F-mCherry. **(A)** Primary structure of human SWAP-70 with mutation L544F indicated. **(B)** Representative maximum projections of confocal Z-stacks for EGFP, mCherry, and wild-type and mutated SWAP-70 localization. Scale bars 4 μm. **(C)** The quantification of protein localization revealed slightly enriched localization of wt SWAP-70 and the clear accumulation of SWAP-70-L544F in dendritic spines. Spine-to-dendrite fluorescence intensity ratios: control: 0.98 (*n* = 15 cells, 147 spines), SWAP-70: 1.15 (*n* = 15 cells, 173 spines), SWAP-70-L544F: 1.78 (*n* = 18 cells, 180 spines). Center lines show the medians; box limits indicate the 25th and 75th percentiles as determined by R software; whiskers extend 1.5 times the interquartile range from the 25th and 75th percentiles, outliers are represented by dots; crosses represent sample means. **p < 0.01, ***p < 0.001 as determined by one-way ANOVA test with Bonferroni *post-hoc* test. **(D)** Maximum projections of confocal Z-stacks of dendrites from neurons overexpressing EGFP along with either mCherry, SWAP-70-mCherry or SWAP-70-L544F-mCherry. Only EGFP channel is shown and was used to assess spine density and morphology. Control image is re-used from Figure 2C. Scale bar, 4 μm. **(E)** Quantification of dendritic spine density calculated as number of spines per 1 μm of dendrite. The first left cluster of bars represents total spine density. Densities of thin, mushroom, stubby, and total spines were as follows: wt: thin = 0.19, mushroom = 0.16, stubby = 0.04, total = 0.39 spines/μm (*n* = 15 neurons, 753 spines, 1,994 μm dendrite); SWAP-70: thin = 0.25, mushroom = 0.18, stubby = 0.03, total = 0.46 spines/μm (*n* = 16 neurons, 762 spines, 1,780 μm dendrite); SWAP-70-L544F: thin = 0.31, mushroom = 0.18, stubby = 0.05, total = 0.54 spines/μm (*n* = 20 neurons, 1,403 spines, 2,672 μm dendrite). Data is pooled from 2 experiments and represented as mean ±SEM. *p < 0.05, **p < 0.01 as determined by one-way ANOVA test with Bonferroni *post-hoc* test. **(F)** Cumulative distributions of the width, length and the ratio of width to length of dendritic spines for neurons expressing either mCherry (control), SWAP-70-mCherry or SWAP-70-L544F-mCherry. Curves are a combination of data points each representing an individual spine. Matching tail regions of the curves are not shown. *p < 0.05, **p < 0.01, ***p < 0.001 as determined by pairwise two-sample Kolmogorov-Smirnov test.

Whole exome sequencing of families with an ASD child revealed a *de novo* missense mutation A/T in *SWAP70*, leading to an amino acid change of leucine (L) 544 to phenylalanine (F) in the C-terminus of SWAP-70 (Figure 11A). The C-terminus of SWAP-70 (amino acids 525–585) is known to be critical for actin binding (Ihara et al., 2006), so a mutation in this region could putatively affect the protein's actin binding properties.

**Figure 12:**
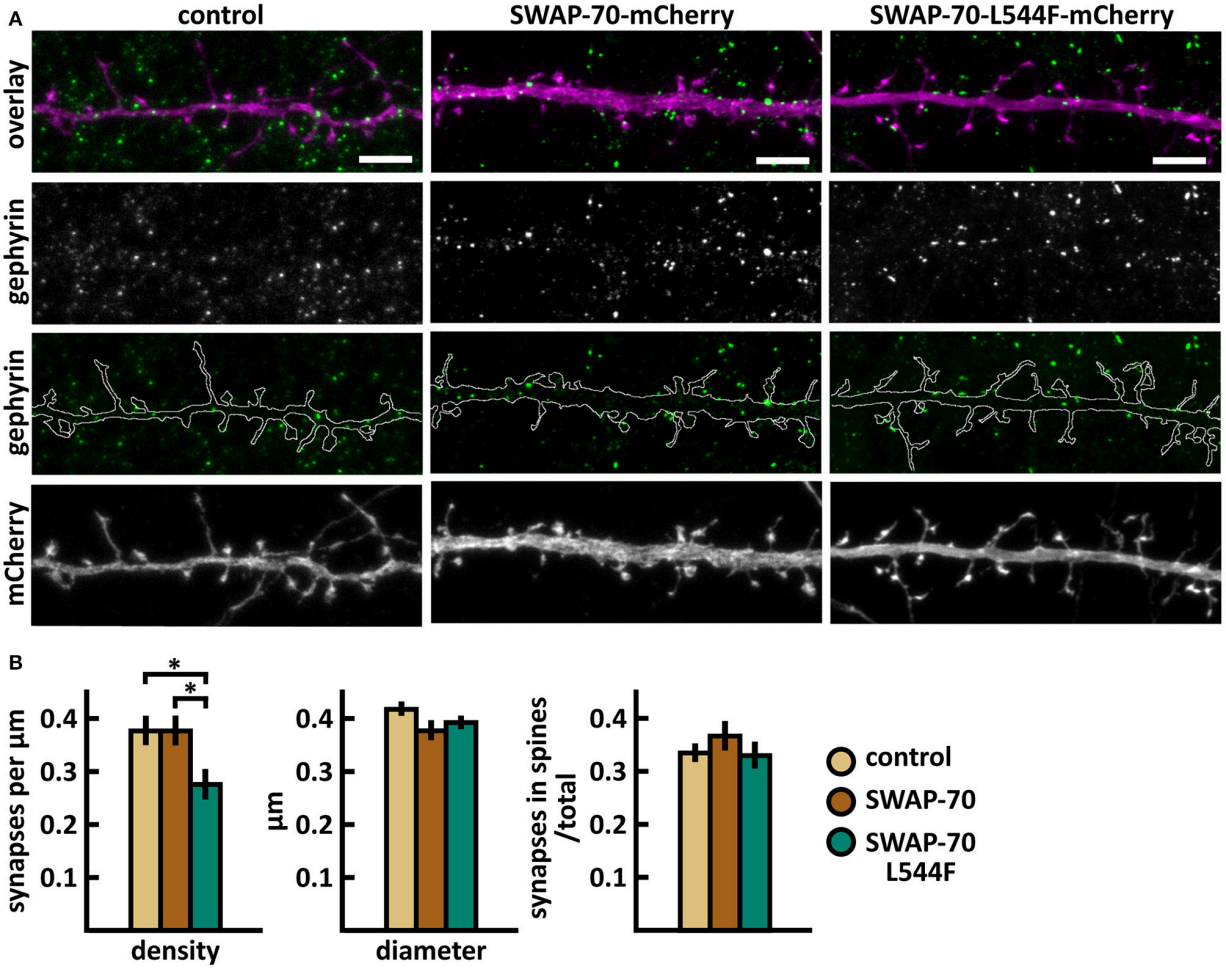
Characterization of the inhibitory synapses in DIV15 hippocampal neurons expressing either mCherry (control), wild-type SWAP-70-mCherry or SWAP-70-L544F-mCherry and stained with anti-gephyrin antibody. **(A)** In overlay, mCherry is shown in magenta and anti-gephyrin antibody staining in green. The line indicates the contour of dendrites (from mCherry). Scale bars, 5 μm. **(B)** Quantification of inhibitory synapse properties. From left to right: (1) Mean density of synapses calculated as the number of gephyrin puncta per μm of the dendrite: control = 0.38; SWAP-70 = 0.38; SWAP-70-L544F = 0.28. (2) Mean diameter of individual gephyrin puncta in μm: control = 0.42; SWAP-70 = 0.38; SWAP-70-L544F = 0.40 3. Proportion of the gephyrin puncta located on dendritic spines as a fraction of the total number of puncta: control = 0.34; SWAP-70 = 0.37; SWAP-70-L544F = 0.34. Data in B represent n(control) = 21 cells, n(SWAP-70) = 23 cells, n(SWAP-70-L544F) = 26 cells, pooled from 4 experiments. ^*^p < 0.05 as determined by one-way ANOVA with Bonferroni's *post-hoc* test. Data is represented as mean ±SEM.

Wild-type SWAP-70 was slightly enriched in dendritic spines when expressed as an mCherry fusion protein in neurons (Figures 11B,C). SWAP-70 L544F-mutation significantly enhanced the spine localization (Figures 11B,C, Table 2). Spine analysis showed increased total and thin spine density for SWAP-70 expressing neurons. The L544F-mutation enhanced these effects, and the difference between cells expressing mutated SWAP-70 and cells expressing mCherry was statistically significant (Figures 11D,E). On average, spine protrusion lengths or head widths in SWAP-70-overexpressing cells were not different from control. However, with the introduction of the mutant SWAP-70-L544F, dendritic spines became thinner (average head width of 0.40 ± 0.01 μm for the mutant vs. 0.44 ± 0.01 μm for wild-type, *p* < 0.05) compared to wild-type SWAP-70. In addition, the distribution of spine morphology parameters showed a similar change toward an increased proportion of thin and short spines (Figure 11F). The density of inhibitory synapses was significantly decreased in mutant-overexpressing neurons compared to both control and wild type-overexpressing neurons (Figures 12A,B, Table 2).

Taken together, wild-type SWAP-70 was neither enriched in dendritic spines, nor did its overexpression alter dendritic spine density or morphology. Wild-type SWAP-70 overexpression did not affect inhibitory synapses either. In contrast, mutated SWAP-70 was enriched in dendritic spines and its overexpression changed the morphology of spines, making them narrower. It also reduced the density of inhibitory synapses by almost 30% compared to wild-type expressing cells (Figure 12B). We conclude that the SWAP-70-L544F mutation changes its function from a non-synaptic regulator to a regulator of dendritic spines and inhibitory synapses.

### SrGAP3 mutant E469K expression enhances localization to spines and increases the proportion of inhibitory synapses in spines

**Figure 13:**
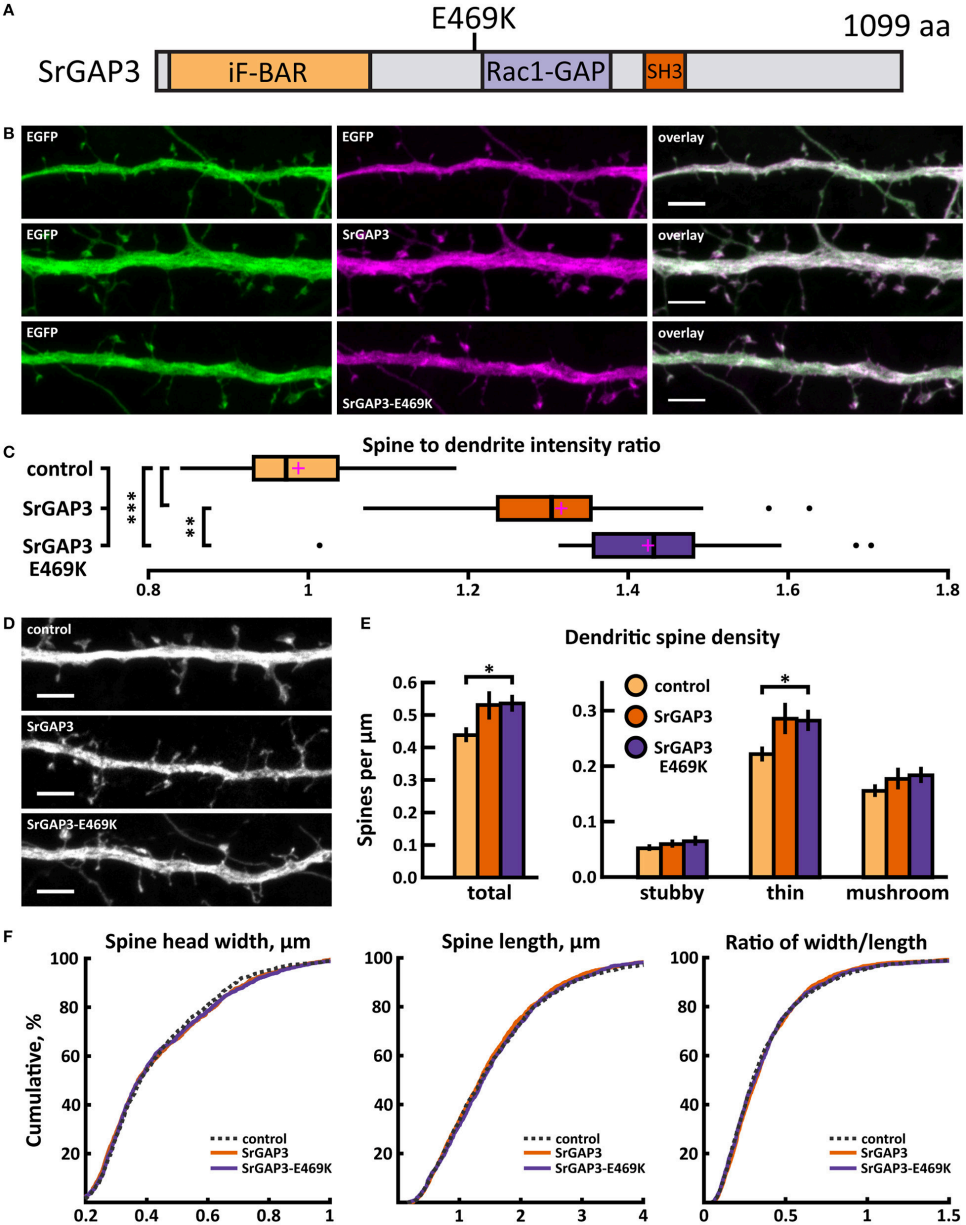
Characterization of protein localization, dendritic spine density and morphology in DIV14-15 hippocampal neurons expressing EGFP and mCherry (control), wild-type SrGAP3-mCherry or mutated SrGAP3-E469K-mCherry. **(A)** Primary structure of human SrGAP3 with mutation E469K indicated. **(B)** Representative maximum projections of confocal Z-stacks for EGFP, mCherry, and wild-type and mutated SrGAP3 localization. Scale bars 4 μm. **(C)** The quantification of protein localization revealed similar localization of both wt and mutated SrGAP3 to dendritic spines. Spine-to-dendrite fluorescence intensity ratios: control: 0.99 (*n* = 34 neurons, 337 spines), SrGAP3: 1.32 (*n* = 25 neurons, 257 spines), SrGAP3-E469K: 1.42 (*n* = 23 neurons, 229 spines). Center lines show the medians; box limits indicate the 25th and 75th percentiles as determined by R software; whiskers extend 1.5 times the interquartile range from the 25th and 75th percentiles, outliers are represented by dots; crosses represent sample means. **p < 0.01, ***p < 0.001 as determined by one-way ANOVA test with Games-Howell *post-hoc* test. **(D)** Maximum projections of confocal Z-stacks of dendrites from neurons overexpressing EGFP along with either mCherry, SrGAP3-mCherry or SrGAP3-E469K-mCherry. Only EGFP channel is shown and was used to assess spine density and morphology. Scale bar, 4 μm. **(E)** Quantification of dendritic spine density calculated as number of spines per 1 μm of dendrite. The first left cluster of bars represents total spine density. Densities of thin, mushroom, stubby, and total spines were as follows: wt: thin = 0.23, mushroom = 0.16, stubby = 0.06, total = 0.45 spines/μm (*n* = 34 neurons, 1,459 spines, 3,401 μm dendrite); SrGAP3: thin = 0.29, mushroom = 0.19, stubby = 0.07, total = 0.54 spines/μm (*n* = 25 neurons, 1,431 spines, 2,792 μm dendrite); SrGAP3-E469K: thin = 0.28, mushroom = 0.19, stubby = 0.07, total = 0.54 spines/μm (*n* = 23 neurons, 1,087 spines, 2,025 μm dendrite). Data is pooled from 3 experiments and represented as mean ±SEM. *p < 0.05 as determined by one-way ANOVA test with Games-Howell *post-hoc* test. **(F)** Cumulative distributions of the width, length and the ratio of width to length of dendritic spines for neurons expressing either mCherry (control), SrGAP3-mCherry or SrGAP3-E469K-mCherry. Curves are a combination of data points each representing an individual spine. Matching tail regions of the curves are not shown. Significant differences were not detected by pairwise two-sample Kolmogorov-Smirnov test.

A *de novo* C/T missense variant in the *SRGAP3* gene was identified in an ASD proband (Sanders et al., 2012). In the SrGAP3 protein, this mutation changes glutamic acid-469 to lysine (E469K), thus changing the charge of the amino acid (Figure 13A).

**Figure 14:**
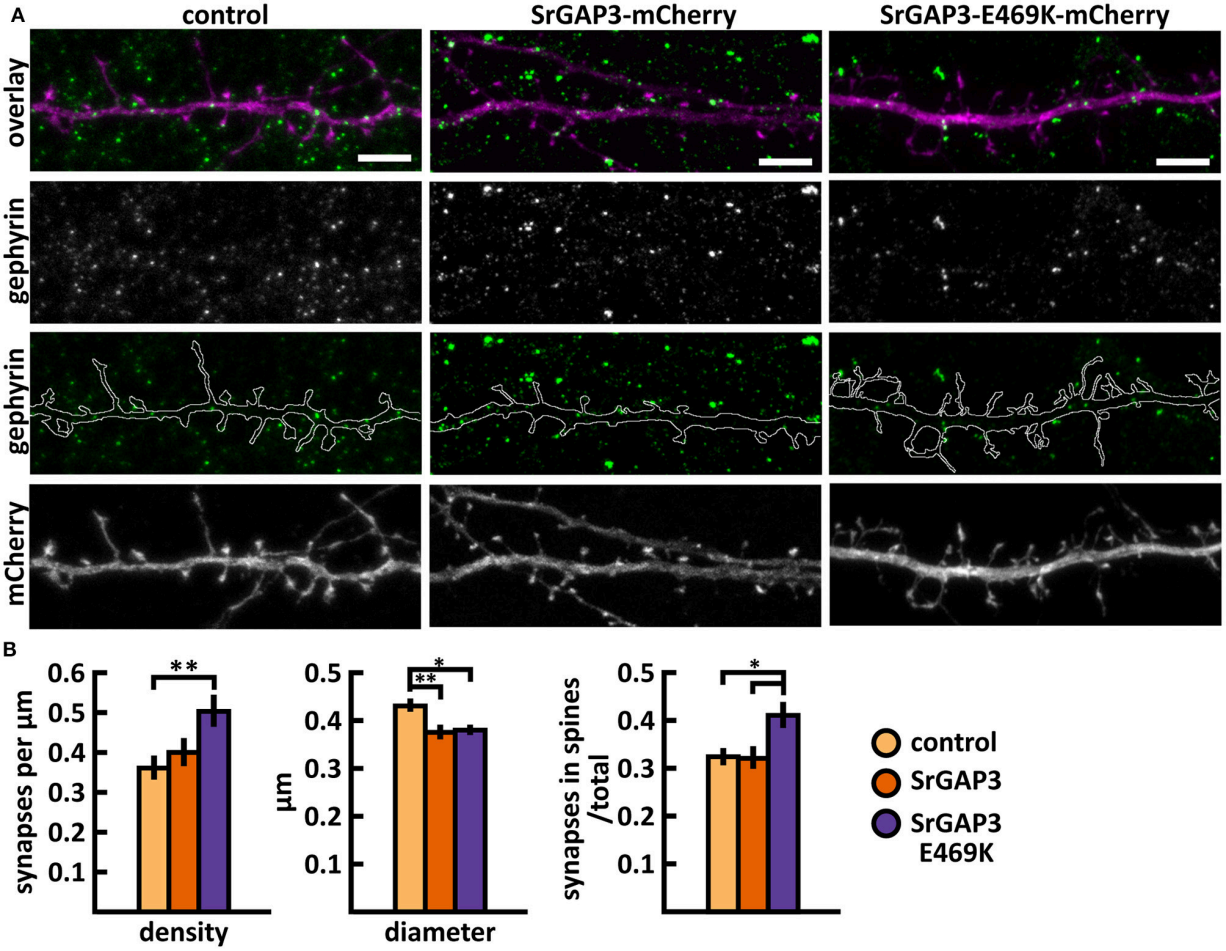
Characterization of the inhibitory synapses in DIV15 hippocampal neurons expressing either mCherry (control), wild-type SrGAP3-mCherry or SrGAP3-E469K-mCherry and stained with anti-gephyrin antibody. **(A)** In overlay, mCherry is shown in magenta and anti-gephyrin antibody staining in green. The line indicates the contour of dendrites (from mCherry). Scale bars, 5 μm. **(B)** Quantification of inhibitory synapse properties. From left to right: (1) Mean density of synapses calculated as the number of gephyrin puncta per μm of the dendrite: control = 0.37; SrGAP3 = 0.41; SrGAP3-E469K = 0.51. (2) Mean diameter of individual gephyrin puncta in μm: control = 0.44; SrGAP3 = 0.38; SrGAP3-E469K = 0.39. 3. Proportion of the gephyrin puncta located on dendritic spines as a fraction of the total number of puncta: control = 0.33; SrGAP3 = 0.33; SrGAP3-E469K = 0.42. Data in B represent n(control) = 17 cells, n(SrGAP3) = 19 cells, n(SrGAP3-E469K) = 18 cells, pooled from 3 experiments. *p < 0.05 and ^**^p < 0.01 as determined by one-way ANOVA with Bonferroni's *post-hoc* test. Data is represented as mean ±SEM.

The mutated SrGAP3 construct enhanced the dendritic spine localization of SrGAP3 (Figures 13B,**C**). Spine analysis showed a significant increase in the density of total and thin spines in neurons that overexpressed SrGAP3-E469K compared to control neurons (Figures 13D,E). We did not observe any difference in the distribution of head widths, lengths, or width-to-length ratios between all 3 groups (Figure 13F). SrGAP3 wild-type expression decreased the size of inhibitory synapses, whereas the expression of mutated SrGAP3 increased the proportion of inhibitory synapses in spines compared to wild-type SrGAP3 expression (Figures 14A,B).

These results show that the E469K mutation enhances the localization of SrGAP3 to dendritic spines. Both wild-type and mutated SrGAP3 increased the density of total and thin spines, and the change between the control and mutated-SrGAP3 groups was statistically significant. The mutation also changed the location of inhibitory synapses by increasing their ratio in dendritic spines.

## Discussion

Both genetic and anatomical studies suggest an important role for defective synapse or spine regulation in ASD. Many actin-modulating proteins known to regulate dendritic spine morphology and density are associated with ASD (Joensuu et al., 2017). However, current knowledge about the mutational effects of these proteins is poor. This has hampered the ability to deduce the prevalence of common defects or cellular phenotypes across different genes and mutations. Based on current literature, we concluded that dendritic spines and inhibitory synapses are good parameters to test the functional consequences of mutations. Dendritic spines are especially suitable because three of the proteins studied (α-actinin-4, myosin IIb, and SrGAP3) have known functions in regulating dendritic spine density and morphology (Zhang, 2005; Ryu et al., 2006; Rex et al., 2010; Carlson et al., 2011; Hodges et al., 2011; Kalinowska et al., 2015). The selection of these parameters was also favored by the fact that both dendritic spines and inhibitory synapses are relatively easy to analyze and could therefore be used for high-throughput screening in future studies. Synaptic transmission would have been another parameter to evaluate, but because the depletion and overexpression of α-actinin-4 resulted in no change in basal synaptic transmission (Kalinowska et al., 2015), we estimated that it is more plausible to detect changes in dendritic spine morphology.

The proper localization of proteins is critical for their correct functioning. Thus, we first analyzed the localization of wild-type and mutated proteins (results summarized in Table 2). Common for all the proteins studied was the enrichment in dendritic spines, suggesting that they can have a role in the regulation of dendritic spine number or morphology. Localization analysis confirmed earlier published results for α-actinin-4 (Kalinowska et al., 2015), myosin IIb (Korobova and Svitkina, 2010; Rubio et al., 2011), and SrGAP3 (Carlson et al., 2011). The mutations induced changes in the localization of α-actinin-4, which localized less to dendritic spines, and for SWAP-70 and SrGAP3, which localized more to dendritic spines. These results show that single amino acid changes can affect the subcellular localization of proteins and a mutation can either reduce (loss-of-function) or induce (gain-of-function) a specific localization.

Next, we studied whether these mutations affect the proteins' overexpression effects on dendritic spine density and morphology in primary hippocampal neurons. Among the wild-type proteins studied, only α-actinin-4 overexpression caused a significant change in dendritic spine morphology. This result confirms the earlier reported spine phenotype (Kalinowska et al., 2015). The spine phenotypes of autism-linked proteins have been variable, but, for example, Shank3 overexpression results in an increase in mushroom spines and in spine head width, similar to α-actinin-4 (Durand et al., 2012). Based on the results of spine analysis, together with published results, we conclude that wild-type α-actinin-4 (Kalinowska et al., 2015), myosin IIb (Zhang, 2005; Ryu et al., 2006; Rex et al., 2010; Hodges et al., 2011), and SrGAP3 (Carlson et al., 2011) have a clear function in regulating dendritic spine density and morphology (summarized in Table 2). Although wild-type myosin IXb and SWAP-70 seem to not have a big impact on spines, this is the first report testing their possible roles in dendritic spines.

We hypothesized that mutations associated with ASD shift dendritic spine morphology from mushroom spines to thin spines. α-actinin-4-M554V mutation resulted in the expected shift in dendritic spine morphology by increasing the thin spine density. This change is similar to what was observed upon the overexpression of ASD-associated Shank3 mutants, which resulted in mostly thinner spines (Durand et al., 2012). In addition to significant changes with the ACTN4 mutation, we saw a trend toward more thin spines with mutations in myosin IXb and SWAP-70 when compared to wild-type proteins. SWAP-70-L544F enhanced the induced increase in thin spine density by the wild-type protein, and the difference between controls and mutation constructs was statistically significant. The increase in thin spines is well supported by the fact that SWAP-70-L544F also increased total spine density and decreased total spine head size, compared to controls. Total spine head size was significantly decreased even when the results were compared to wild-type SWAP-70. Taken together, although most of the changes were mild and the differences between wild-type and mutated proteins were not always significant, we observed a trend toward an increased proportion of thin spines.

Current literature suggests that, compared to bigger mushroom spines, thin spines are structurally more dynamic and transient (Kasai et al., 2003; Holtmaat et al., 2005). Thin spines have weaker synapses than mushroom spines, but they are more susceptible to potentiation (Matsuzaki et al., 2004). Mushroom spines with large heads exhibit more AMPA receptors and stronger excitatory postsynaptic responses (Matsuzaki et al., 2001), and are stable *in vivo* over months (Grutzendler et al., 2002; Trachtenberg et al., 2002; Holtmaat et al., 2005). These correlations have led to the proposal that the small dynamic spines are preferentially involved in learning, whereas larger stable spines mediate long-term memory storage (Kasai et al., 2003). Thus, it is probable that the most frequently observed effect of the mutations—the increased density of thin spines—also affects neuronal function and behavior. Accordingly, studies on Fragile-X syndrome (FXS) and *Shank1* ASD-mouse models have shown a correlation between spine morphology, neuron functionality, and behavior, supporting the importance of proper dendritic spine morphology and density for normal synaptic plasticity and behavior. In the *Fmr1*-deficient FXS mouse model, dendritic spines are thin and elongated and the density is increased (Comery et al., 1997). Neurons of *Fmr1* KO mice exhibit altered synaptic plasticity (Huber et al., 2002; Zhao et al., 2005; Nosyreva and Huber, 2006). In the *Fmr1* KO synapses, a lower ratio of AMPA to NMDA receptors was detected early in development compared to wildtype controls (Pilpel et al., 2009). These data demonstrate that the lack of Fmr1 produces alterations in normal synaptic activity, which likely contributes to the FXS phenotype. Behavioral analyses have revealed autism-related behavior, such as hyperactivity, repetitive behaviors, and seizures (reviewed in Kazdoba et al., 2014). By rescuing the spine density or morphology, autism-related behavior was rescued (Dolan et al., 2013; Pyronneau et al., 2017). *Shank1*-KO mice showed thinner dendritic spines, smaller excitatory synapses, and weaker basal synaptic transmission (Hung et al., 2008). In contrast to FXS mice, synaptic plasticity was normal in these mice. Behaviorally, they had increased anxiety-related behavior and impaired contextual fear memory (Hung et al., 2008). In line with the idea that small dynamic spines are preferentially involved in learning, whereas larger stable spines mediate long-term memory storage (Kasai et al., 2003), *Shank1*-deficient mice displayed enhanced performance in a spatial learning task but their long-term memory was impaired (Hung et al., 2008).

Finally, we analyzed the size, density, and localization of inhibitory synapses. In addition to increasing our knowledge of the effects of ASD-mutations, we provide here new information for whether actin binding proteins in general affect inhibitory synapses. This is still a very poorly characterized field and these results may open new avenues for actin regulation of inhibitory synapses. Changes in inhibitory synapses varied between the proteins studied. Myosin IIb and myosin IXb increased the proportion of inhibitory synapses in spines, whereas SrGAP3 decreased the size of inhibitory synapses. Thus, it seems that actin-binding proteins can affect various parameters in inhibitory synapses but the detailed molecular mechanisms are still open. In the future, it will also be interesting to see whether the spiny localization of inhibitory synapses will affect the inhibition efficiency or modality of the synapses. Currently, it seems that positions of shaft inhibitory synapses determine the hotspots of synapse remodeling, where both new spines and new inhibitory synapses are more likely to be formed (Chen et al., 2012; Isshiki et al., 2014). Furthermore, inhibitory synapses in spines seem to stabilize spines (Isshiki et al., 2014). In line with this result, the loss of inhibitory synapses from spines could indicate an increased turnover rate of spines. This is interesting in the context of ASD as an increased turnover of spines was found to be a common parameter for two tested ASD-mouse models (Isshiki et al., 2014). Here, the expression of mutated myosin IIb (Y265C) reduced, but mutated SrGAP3 (E469K) increased, the proportion of inhibitory synapses in spines (Table 2). In addition, α-actinin-4, myosin IXb, and SWAP-70 mutations showed a trend toward a reduced proportion of spiny inhibitory synapses. Furthermore, the expression of myosin IIb-Y265C decreased the inhibitory synapse size and SWAP-70-L544F expression decreased the density of inhibitory synapses.

It is important to note that although we did not observe changes in dendritic spines or inhibitory synapses for all mutations, it is possible that the mutations do affect synaptic transmission and behavior. The effects of the mutations are obviously not restricted to synapses, but mutations can affect other cellular processes in the brain, from dendrite growth to the various functions of glial cells. Functional defects of glial cells are implicated in ASD (Petrelli et al., 2016) and, in fact, all genes studied here are expressed in higher levels in human astrocytes than in neurons (Zhang et al., 2014).

With overexpression analysis in cultured neurons we can only determine whether a mutation changes the protein's function. Proper evaluation of the contribution of a mutation to the development of autism requires knock-in animal studies. A very nice example of this is a thorough evaluation of *SHANK3* ASD and schizophrenia mutations in mouse models showing both shared and distinct defects in synaptic transmission, behavior, and spine density (Zhou et al., 2016). However, these type of studies are time-consuming and require plenty of resources. Therefore, the pre-screening of mutations is necessary to pre-select mutations for detailed studies. Furthermore, our screening-type experiments give a broader view on the impact of *de novo* missense mutations and which cellular parameters should and could be used as readouts for protein functionality. From these five genes, only the α-actinin-4 mutation showed a substantial effect on dendritic spines and should be taken to further animal studies. The effects of all the other mutations were relatively mild, but it is possible that under suitable circumstances, possibly enhanced by other mutations or risk-variants, these mutations can contribute to the development of autism.

## Author contributions

IH carried out the experiments and analyses for Figures 2, 6, 9, 11, 13; PK was responsible for inhibitory synapse experiments and analyses presented in Figures 3, 4, 7, 10, 12, 14. AA designed and cloned all mutation constructs. VP generated the protein structures for Figures 1, 5, 8 and placed the hypotheses of the functional consequences of different mutations. PH conceived the theoretical ideas in this work, drew final conclusions and led the writing of the manuscript. IH, PK, AA, and VP wrote sections of the manuscript. All authors contributed to writing and editing of the manuscript. IH was responsible for the final layout of the Figures.

### Conflict of interest statement

The authors declare that the research was conducted in the absence of any commercial or financial relationships that could be construed as a potential conflict of interest.
